# Study of blood linear RNA nominates *CD55* and *DLD* as early-stage biomarkers for Parkinson’s sisease

**DOI:** 10.1093/brain/awag014

**Published:** 2026-10-05

**Authors:** Aleksandra Beric, Sarp Sahin, Santiago Sanchez, Zining Yang, Ravindra Kumar, Isabel Alfradique-Dunham, Jessie Sanford, Daniel Western, Bridget Phillips, John P. Budde, Richard J. Perrin, Paul T. Kotzbauer, Joel S. Perlmutter, Scott A. Norris, Carlos Cruchaga, Laura Ibanez

**Affiliations:** 1 Department of Psychiatry, Washington University in Saint Louis School of Medicine, St. Louis, MO 63110, USA; 2 NeuroGenomics and Informatics Center, Washington University in Saint Louis School of Medicine, St. Louis, MO 63110, USA; 3 Department of Neurology, Washington University in Saint Louis School of Medicine, St. Louis, MO 63110, USA; 4 Department of Pathology & Immunology, Washington University in Saint Louis School of Medicine, St. Louis, MO 63110, USA; 5 Department of Radiology, Washington University in Saint Louis School of Medicine, St. Louis, MO 63130, USA; 6 Department of Neuroscience, Washington University in Saint Louis School of Medicine, St. Louis, MO 63110, USA; 7 Program of Physical Therapy, Washington University in Saint Louis School of Medicine, St. Louis, MO 63108, USA; 8 Program of Occupational Therapy, Washington University in Saint Louis School of Medicine, St. Louis, MO 63108, USA; 9 Hope Center for Neurological Disorders, Washington University in Saint Louis School of Medicine, St. Louis, MO 63110, USA; 10 The Charles F. and Joanne Knight Alzheimer Disease Research Center, Washington University in Saint Louis, St. Louis, MO 63108, USA; 11 Department of Genetics, Washington University in Saint Louis School of Medicine, St. Louis, MO 63110, USA

**Keywords:** Parkinson’s disease, blood transcriptomics, CD55, DLD, biomarkers

## Abstract

Parkinson’s disease is the second leading neurodegenerative disease, pathologically characterized by the accumulation of alpha-synuclein in the brain and the loss of dopaminergic neurons in the substantia nigra pars compacta. Despite the intensive efforts to identify diagnostic biomarkers for Parkinson’s disease nominating prospective candidates such as such as CSF α-synuclein seed amplification assay or α-/β-synuclein ratio, establishment of minimally invasive biomarkers remains the focus of Parkinson’s disease research.

We leveraged transcriptomic data from 4,343 participants from four independent datasets to robustly identify Parkinson’s disease-associated transcripts. Following the differential abundance analyses, we integrated our findings with several brain transcriptomic, CSF proteomic, plasma proteomic and genomic data to add biological context. We further leveraged our findings to develop predictive models that could differentiate between Parkinson’s disease and healthy controls.

We identified 296 differentially expressed transcripts, 28 of which were transcribed from known Parkinson’s disease-associated loci. Further, we found a significant overlap between our findings and transcripts dysregulated in brain, as well as proteins differentially accumulated in CSF. Our results suggest that expression of the identified transcripts was affected by genetic background including ancestry and Parkinson’s disease-related mutations, and nearly half of the identified transcripts were dysregulated before symptom onset. The differentially expressed transcripts were utilized to develop three predictive models that distinguished between Parkinson’s disease and healthy controls with a ROC AUC of 0.727–0.733. The predictive models were capable of detecting Parkinson’s disease transcriptomic signatures even before symptom onset. Overall, two transcripts, *DLD* and *CD55*, showed particular promise as early stage, minimally invasive Parkinson’s disease biomarkers. *DLD* significantly related to Parkinson’s disease in the eQTL analyses and we identified a suggestive eQTL for *CD55,* their protein products were differentially accumulated in CSF, and both *DLD* and *CD55* were included in all three predictive models. Thus, we have performed the largest Parkinson’s disease transcriptomic study to date and demonstrated that the transcriptome can be leveraged for development of minimally invasive biomarkers that could aid in diagnosing Parkinson’s disease.

## Introduction

Parkinson’s disease is a progressive neurodegenerative disease characterized by motor (e.g. resting tremor, rigidity, postural instability, bradykinesia), and non-motor features (e.g. cognitive decline, depression, pain).^1,2^ Affecting 2–3% of the population over 65, Parkinson’s disease is the second-most common neurodegenerative disorder and its prevalence may double between 2015 and 2040.^3,4^ Parkinson’s disease is pathologically defined by the accumulation of misfolded and abnormally phosphorylated alpha-synuclein (α-Syn) protein in Lewy bodies and neurites within the brain.^5^ This protein aggregation occurs alongside degeneration of dopamine-producing neurons in the substantia nigra, a critical brain region for movement control.^5^ Current therapeutic strategies focus on managing Parkinson’s disease symptoms, but no current therapy prevents neuronal degeneration or slows disease progression. A deeper understanding of the underlying mechanisms driving Parkinson’s disease pathogenesis is imperative to develop more effective treatments which could include gene-targeted therapies.

Blood transcriptomics offers a compelling approach to investigate Parkinson’s disease pathogenesis. Compared to invasive tissue biopsies or expensive imaging procedures, blood draws are minimally invasive and readily available in most healthcare settings. Moreover, whole blood, which shares over 80% overlap with neuronal gene expression, holds potential for capturing meaningful molecular changes at the transcript level.^6–9^ Linear RNA, in particular, reflects actively transcribed genes within cells, providing a real-time snapshot of cellular processes. This dynamic view of cellular activity has been leveraged in previous Parkinson’s disease transcriptomic studies to consistently identify disease-associated genes, transcripts, and enriched pathways.^10^ For instance, a study measuring differences in blood RNA expression data identified and validated eight genes associated with Parkinson’s disease risk from a recent meta-analysis of genome-wide association studies (GWAS).^11,12^ Further supporting the utility of blood-based analysis, Irmady et al. identified 1,584 transcripts that were significantly dysregulated in Parkinson’s disease blood and showed similar changes in expression within the caudate and putamen regions of the Parkinson’s disease brain.^13^ Makarious *et al*. integrated whole blood RNA-seq data with clinical, demographic and genome sequencing data to develop a multi-modal model capable of predicting Parkinson’s disease with 85.03% accuracy and 93.12% sensitivity, demonstrating that the blood transcriptome captures biologically significant changes that, combined with other data modalities can improve the accuracy of Parkinson’s disease diagnosis.^14^ However, the generalizability of most of the transcriptomic findings has been limited by relatively small sample sizes and use of different methodologies.

The search for diagnostic biomarkers for Parkinson’s disease has intensified in recent years, driven by the promise of earlier detection and intervention. While recent studies proposed some promising Parkinson’s disease biomarker candidates, such as CSF α-synuclein seed amplification assay,^15^ identification of abnormal α-synuclein on skin biopsies and α-/β-synuclein ratio,^16^ identification of non-invasive Parkinson’s disease biomarkers remains paramount as it would greatly aid disease diagnosis and monitoring.

To address this gap in knowledge, we conducted a comprehensive analysis of whole blood transcriptomic profiles in the largest Parkinson’s disease cohort to date. We hypothesized that blood transcripts can be leveraged as reliable Parkinson’s disease biomarkers and utilized to develop predictive models capable of distinguishing Parkinson’s disease from healthy controls with high accuracy. We have processed and meta-analyzed the transcriptomic profiles from four major studies: the Parkinson’s Disease Biomarkers Program (PDBP),^17^ the Parkinson’s Progression Markers Initiative (PPMI),^18^ the Fox Investigation for New Discovery of Biomarkers (BioFIND),^19^ and Washington University in St. Louis Movement Disorders Clinic cohort (WashU-MDC). Then, we performed pathway analyses and multi-omic data integration to understand the biological implication of our findings. We identified if the transcripts are dysregulated in brain and if the protein products are dysregulated in plasma and/or CSF of Parkinson’s disease participants. Next, we examined whether those transcripts were encoded in known Parkinson’s disease-associated genetic loci, identified cis-expression quantitative trait loci (eQTL) and performed Mendelian Randomization (MR) to infer causality. Finally, we assessed the predictive power of the blood transcriptome by leveraging it to build preliminary biomarkers for disease detection and follow-up. Unlike previous research limited by small sample sizes and varying methodologies, our approach leverages meta-analysis, pathway analyses, and multi-omic data integration to robustly identify dysregulated transcripts and propose them as potential non-invasive Parkinson’s disease biomarkers. By assessing the predictive power of these transcripts for disease detection and follow-up, this study aims to advance the understanding of Parkinson’s disease pathogenesis and pave the way for novel therapeutic strategies.

## Materials and methods

### Study design

**Figure 1:**
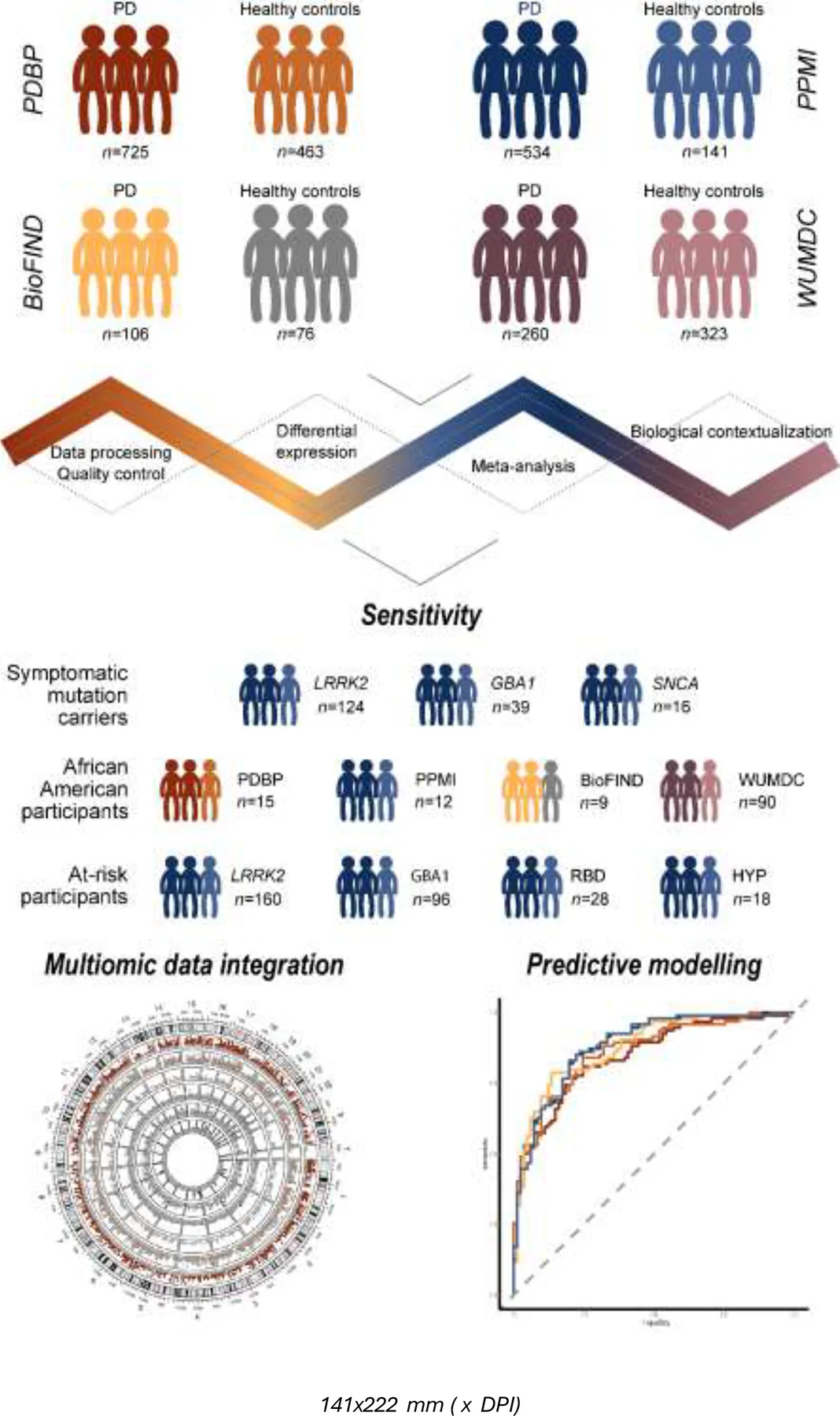
Study design.

We leveraged four independent Parkinson’s disease whole blood RNAseq datasets: PDBP^17^ (*N*=1,604), PPMI^18^ (*N*=1,943), BioFIND^19^ (*N*=213), and WashU-MDC^20^ (*N*=583; Figure1). We performed differential expression (DE) analyses in each cohort separately and then combined the findings from all four cohorts via meta-analysis. Only transcripts with the same direction of effect in all four cohorts were assessed in the meta-analysis, and those with multiple-test corrected p-values lower than 0.05 in the meta-analysis were considered DE. Next, we biologically contextualized our findings through pathway analyses and integration with an in-house brain transcriptomic and publicly available plasma and CSF proteomic datasets. We also leveraged the available Parkinson’s disease GWAS resources to verify whether any of the DE transcripts originated from known Parkinson’s disease-risk loci. For all significant transcripts, we identified the eQTLs and tested causality using MR. Further, we evaluated the effect that ancestry and Parkinson’s disease-related mutations had on the whole blood transcriptomic landscape. Then, we tested whether there is detectable transcript dysregulation in participants without symptoms, but at risk of developing Parkinson’s disease. We explored clinical importance of our findings by investigating the impact of medication on the expression of the identified transcripts, as well as the relationship of the DE transcripts with UPDRS-III and MoCA scores, measures of motor symptom and dementia severity, respectively. Finally, we leveraged our findings to develop predictive models capable of distinguishing between Parkinson’s disease and healthy control participants. This study was approved by the Washington University in Saint Louis Institutional Review Board (IRB IDs: 201701124 and 202004010).

### Study population

PDBP and PPMI enrolled participants who were followed longitudinally with clinical assessments and biospecimen collection every six months. Blood RNA-seq data was collected every six months in PDBP, and every six months during the first year followed by annually thereafter in PPMI. Instead of using baseline as previous publications^11,21^ have done for cross-sectional analyses, we selected the last assessment of each participant in an attempt to maximize clinical and molecular differences between cases and controls, and harmonize with the BioFIND and WashU-MDC cohorts inclusion criteria. BioFIND and WashU-MDC cohorts employed a cross-sectional study design. A total of 2,628 participants of European descent were included in the main analysis, consisting of 1,625 Parkinson’s disease cases (*PPMI*=534, *PDBP*=725, *BioFIND*=106, *WashU-MDC*=260) and 1,003 control participants (*PPMI*=141, *PDBP*=463, *BioFIND*=76, *WashU-MDC*=323; Table 1). Mean ages of the participants in PDBP (64.65±10.10) did not significantly differ from PPMI (63.85±9.83) (*p*=0.10). However, BioFIND participants, with the mean age of 67.21±6.86, were significantly older than both the PDBP (*p*=1.70×10^−5^) and PPMI (*p*=1.91×10^−7^) participants. Similarly, WashU-MDC participants, whose mean age was 69.21±9.26, were significantly older than participants in either of the other three cohorts, PDBP (*p*<2.20×10^−16^), PPMI (*p*<2.20×10^−16^) and BioFIND (*p*=1.89×10^−3^). Consistent with the well-documented higher prevalence of Parkinson’s disease in males,^22,23^ all four datasets exhibited a comparable higher male proportion (>60%) of Parkinson’s disease participants (*p*=0.72). Healthy controls were balanced by sex in all datasets except PPMI, which had a significantly higher proportion of males (63.57%) than healthy control participants in PDBP (45.14%, *p*=2.70×10^−4^), BioFIND (47.36%, *p*=0.04), or WashU-MDC (43.96%, *p*=2.20×10^−4^). BioFIND Parkinson’s disease participants had significantly higher UPDRS-III scores (39.07±13.23) compared to those from PPMI (24.84±13.15, *p*<0.001) or PDBP (25.83±13.36, *p*<0.001). Healthy control participants had similar mean UPDRS-III scores in PPMI (1.48±2.62) and PDBP (2.12±5.06; *p*=0.72). UPDRS-III scores were not available for WashU-MDC participants or BioFIND healthy control participants. Finally, Parkinson’s disease participants in the PDBP cohort presented with significantly lower MoCA scores (26.62±2.55) compared to those in PPMI (26.36±3.08, *p*=8.30×10^−9^) or BioFIND (26.84±2.57, *p*=3.70×10^−6^). Likewise, PDBP healthy controls participants had significantly lower MoCA scores (26.62±2.55) than either PPMI (26.62±2.55, *p*=3.80×10^−3^) or BioFIND (27.99±1.52, *p*=2.40×10^−5^) healthy control participants. No comparison was performed to the WashU-MDC MoCA scores, due to absence of information.

### Data processing and quality control

We accessed raw transcriptomic data (fastq files) from 7,598 ribodepleted blood RNA samples across the PDBP, PPMI, BioFIND, and WashU-MDC studies. Detailed information on sample collection and data generation for these datasets are described elsewhere.^11,20,24,25^ The publicly available datasets, PDBP, PPMI, and BioFIND, were accessed through the Accelerating Medicines Partnership Parkinson’s Disease (AMP-PD) Knowledge Portal. While all the data processing and quality control (QC) have previously been completed for each of the datasets, we processed and QC-ed the data *de novo* to ensure that the same criteria were applied to all datasets. We processed the raw data using in-house pipelines to generate gene-level expression counts.^20^ Briefly, we aligned the raw reads to the human genome reference (GRCh38) and annotated transcripts with Gencode 33 annotation using STAR (v.2.7.8a).^26^ Alignment quality was assessed via Picard metrics (v.2.25.0)^27^ and gene expression quantified with Salmon (v.1.7.0),^28^ collapsing transcripts to gene level. To maintain high quality standards, we removed from downstream analyses any samples with low mapping rates (<50% of reads mapped in STAR or Salmon), those that failed QC in more than four categories defined by FastQC, or that presented outliers in the Principal Component Analyses (PCA). PCA outliers were deemed those samples whose values of the first or second principal component differed more than three standard deviations from the mean values of those respective principal components. Additionally, transcripts with low expression, described as having fewer than 10 reads in >90% of individuals, were excluded from analyses. Normalization was performed using the variance stabilizing transformation (vst) function from the *DESeq2*^29^ package to adjust for library complexity and generate the count matrices used in PCA.

### Differential abundance analysis and pathway analyses

Differential expression (DE) analyses were performed using *DESeq2 (v 1.44.0)*.^29^ Analyses were carried out on gene-collapsed transcript counts and adjusted by participants’ age at blood collection and sex as well as relevant technical variables.^30^ To account for major confounding factors in blood transcriptomics, such as variation in cellular composition, cell proportions were obtained using deconvolute function from the *immunedeconv*^31^ package and subsequently included in the model. Results of DE analyses from all four cohorts were integrated via meta-analysis using the *metaRNAseq*^33^ package. Only genes exhibiting the same direction of effect across all four datasets were considered for the meta-analysis. Benjamini-Hochberg adjusted p-values lower than 0.05 were considered statistically significant. We performed pathway analyses on the DE genes identified in the meta-analysis using *ClusterProfiler*^34^ and *DOSE*^35^ packages.

### Sensitivity analyses

To examine if the findings were independent of ancestry, we also assessed transcriptomic patterns in 126 participants of African ancestry (*PPMI*=12, *PDBP*=15, *BioFIND*=9, *WashU-MDC*=90; Supplementary Table 1). Due to limited sample size, data from all four cohorts was combined into a single dataset for these analyses. Prior to combining, transcript counts were scaled between the four cohorts by calculating z-scores. Normalized, scaled counts were then fit to a linear regression model to compute the difference in expression levels between Parkinson’s disease and healthy control participants for each transcript.

Next, to determine if Parkinson’s disease associated mutations affect the whole blood transcriptomic landscape, we investigated the expression patterns in 124 *LRRK2*^+^, 39 *GBA1*^+^, and 16 *SNCA*^+^ symptomatic participants (Supplementary Table 1) from PPMI. The most common mutations in the symptomatic participants were G2019S (*n*=90) in *LRRK2*^+^, N409S (*n*=31) in *GBA1*^+^ and A53T (*n*=16) in *SNCA*^+^ participants. Additionally, we explored whether the changes in expression of the DE transcripts start prior to symptom onset by assessing expression in those individuals without symptoms but at risk of developing Parkinson’s disease, such as asymptomatic participants with Parkinson’s disease-associated mutations (160 *LRRK2*^+^ and 96 *GBA1*^+^), as well as participants who exhibited REM sleep behavior disorder (RBD, *n*=28) or hyposmia (*n*=18; Supplementary Table 1). Similar to symptomatic participants, the most common mutation in at-risk *LRRK2*^+^ participants was G2019S (*n*=119), and N409S (*n*=66) in *GBA1*^+^ participants. Detailed mutation carrier information for symptomatic participants, as well as data for at-risk participants, was only available in the PPMI cohort. University of Pennsylvania Smell Identification Test (UPSIT) was used to evaluate olfactory capabilities of the participants, and those with scores below the tenth percentile, relative to age- and sex-matched reference data, were considered hyposmic. Probable RBD was primarily determined using the REM Sleep Behavior Disorder Screening Questionnaire (RBDSQ), with score threshold of five.

### Correlation of DE transcripts and clinically relevant parameters

We used DESeq2 to explore the effect of Parkinson’s disease medication on transcript expression in symptomatic participants. Detailed medication data was available for the PPMI cohort only, limiting these analyses to the 486 participants with levodopa equivalent information.

To explore the clinical relevance of the identified transcripts, we assessed correlations between normalized transcript counts and UPDRS-III or MoCA scores in participants with available data (regardless of diagnosis). We performed linear regression analysis, adjusted by participants’ age and sex. Due to data availability, these analyses were done in PDBP, PPMI and BioFIND cohorts, separately. Afterward, we employed *combinePvalues* function from the multiGSEA (version 1.14.0)^36^ package to calculate the combined p-value via Fisher’s combined probability test. We considered correlations statistically significant at a p-value threshold of 0.05. Further, to determine if transcript levels were causally associated with medication usage and motor symptom severity, we performed mediation analyses using R package mediation.^37^

### Multiomic data integration

To further biologically frame our results, we substantiated our findings through integration with: (i) in-house brain bulk RNAseq, and (ii) publicly available plasma and (iii) CSF proteomic data.^38^ The in-house brain dataset included 137 parietal brain samples, corresponding to 99 Parkinson’s disease and 38 HC participants. After DE of the brain RNAseq, or the differential accumulation analyses of the two proteomic datasets, we evaluated the overlap between nominally significant findings from each of the accessed datasets and the DE transcripts identified in whole blood. We used the hypergeometric test (phyper function in R) to test the significance of each overlap. P values lower than 0.05 were considered significant.

Further, we leveraged the latest Parkinson’s disease genome wide association studies (GWAS)^39,40^ and information compiled in the Parkinson’s disease GWAS locus browser^41^ to query whether any of the identified DE transcripts originated from known Parkinson’s disease associated loci. To compare our results to Parkinson’s disease GWAS, all lead SNP coordinates were converted from hg19 to hg38 version of the human genome. Next, coordinates 500kb upstream and downstream from each SNP were found, and all overlapping regions collapsed into 69 non-overlapping genomic regions. Subsequently, bedtools intersect from the BEDTools tool suite^42^ was used to check for overlap between the collapsed Parkinson’s disease-associated genomic regions and start and end coordinates of the identified DE genes. Finally, we browsed the Parkinson’s disease GWAS locus browser^41^ and recorded the scores, which rank the genes based on the level of support, for those genes that overlapped Parkinson’s disease-associated loci.

### Expression Quantitative Loci (eQTL) mapping

To identify the genetic loci that were involved in the regulation of the identified DE transcripts, we conducted a GWAS specifically on the DE transcripts to identify the eQTLs. We accessed the genotyping information from the WashU-MDC participants^18–20^ and followed standard pipelines.^20^ In brief, all samples were genotyped using Illumina GSA SNP array technology and variants were called using GenomeStudio. Imputation was performed on the TOPMed Imputation Server using the GRCh38 version R2 reference panel. Stringent quality control was applied before and after imputation. In summary, individuals or SNPs with a call rate lower than 98% were removed. Autosomal SNPs that were not in Hardy-Weinberg equilibrium (*P*<1×10^−06^) were also removed. The SNPs from the X chromosome were used to infer sex based on heterozygosity rates. Those samples with discordances between inferred sex and reported sex were removed. Additional QC included removal of palindromic variants, pairwise genome-wide estimates of proportion identity-by-descent to identify and remove unexpected duplicates, and removal of cryptically related samples (*PI-HAT*>0.30). Finally, principal component analysis (PCA) was performed using 1K genomes as an anchor to identify non-Hispanic White participants to keep the population homogeneous as possible for analyses. GWAS was conducted using PLINK2 with a linear regression model.^43^ To account for potential confounding factors, all analyses were adjusted by age, sex and genetic principal components one through five. The genome-wide significance threshold was set at *p*<5´10^−08^. Finally, to assess if the effect size of a genetic variant known to be associated with Parkinson’s disease is mediated by the gene expression of any of the DE transcripts identified in here, or in other words, to infer causality between Parkinson’s disease and the DE transcripts, we used the summary-data-based MR^35^ to test for association between the expression level of a gene and Parkinson’s disease genetic risk using the latest Parkinson’s disease GWAS.

### Predictive model development and evaluation

We leveraged DE transcripts and a previously established pipeline^9,44^ to construct predictive models. Briefly, vst-normalized and age and sex adjusted transcript counts were scaled using z-scores to account for technical differences between the different datasets. Next, we utilized the R package entropy v1.3.1^45^ to compute Kullback-Leibler divergence (KLD) for each transcript in the training (PDBP) compared to the testing (PPMI) dataset. Subsequently, we trained 100 L2 regularization linear models with transcripts ranging in KLD value from 0.01 to 1 in increments of 0.01 and calculated the Area Under the Receiver Operating Characteristic (ROC) Curve (AUC) value for all models in the training dataset. PDBP was used to train, and PPMI to test. WashU-MDC and BioFIND were used to validate the predictive models. Additionally, and only for the PPMI dataset, we evaluated the performance of the selected models in Parkinson’s disease participants with Parkinson’s disease-associated mutations, as well as the ability of the models to differentiate between healthy controls and at-risk participants.

## Results

### Whole blood captures Parkinson’s disease associated transcriptomic patterns

**Figure 2:**
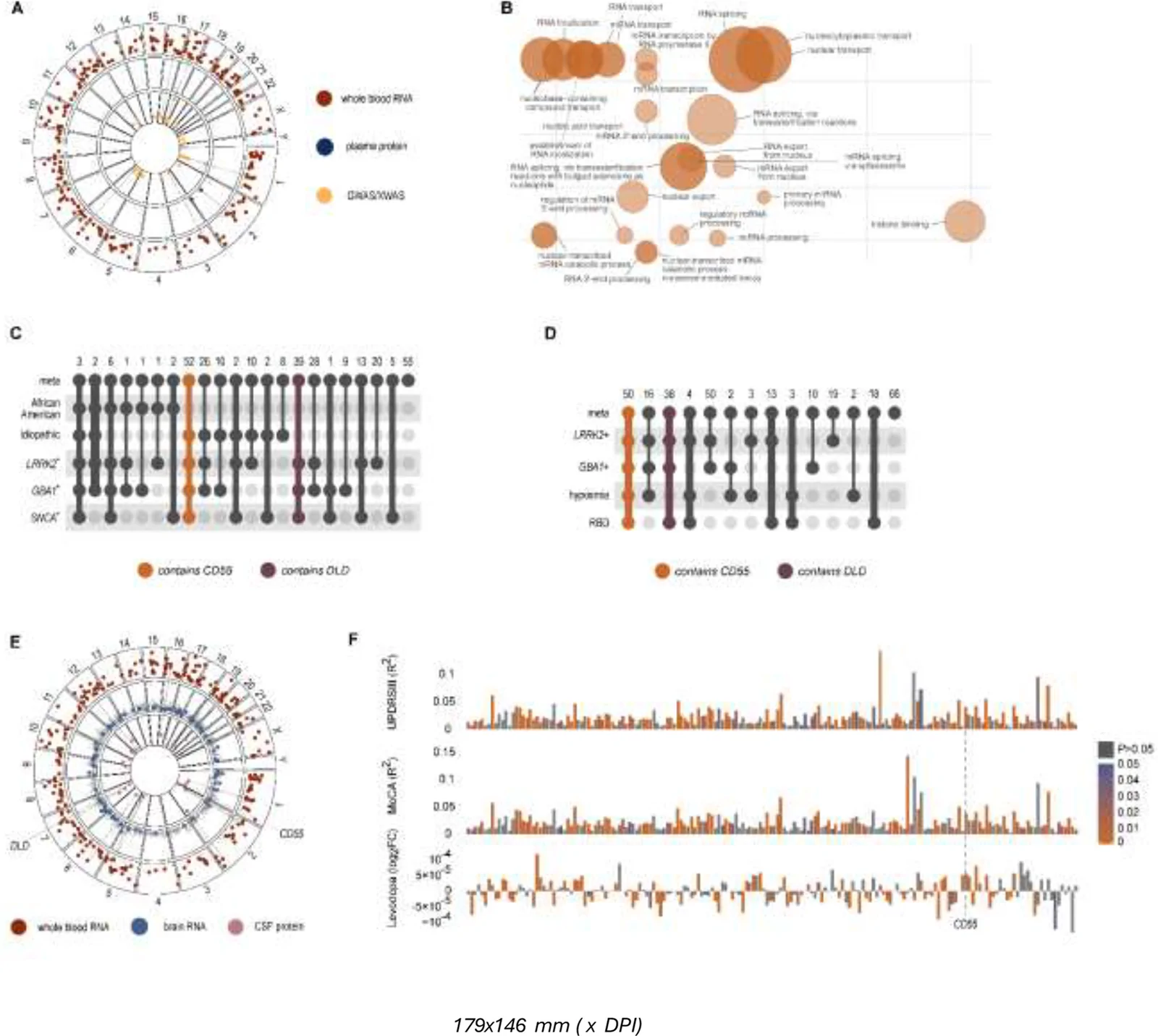
Multi-omic overlap and sensitivity analyses. **(A)** Integration between differential expression analysis of (**i**) whole blood RNAseq (maroon) with plasma proteomics (dark blue) and GWAS data (light yellow); and (**ii**) whole blood RNAseq (maroon) with brain RNAseq (light blue) and CSF proteomics (rosy-brown). **(B)** Scatter plot summarizing the significant results of GO pathway analysis. **(C)** intersection matrix showing results of sensitivity analyses in symptomatic participants. **(D)** Intersection matrix showing results of sensitivity analyses in at-risk participants. (**E**) Histograms showing association between normalized transcript counts and UPDRS-II scores (*top*), MoCA sores (middle), and Levodopa levels (*bottom*).

To identify transcripts that were associated with Parkinson’s disease, we leveraged four independent whole blood transcriptomic datasets, PDBP, PPMI, BioFIND, and WashU-MDC, totaling 1,625 non-Hispanic white Parkinson’s disease participants (*N*_*PDBP*=_725, *N*_*PPMI*_=534, *N*_*BioFIND*_=106, *N*_*WashU-MDC*_=260) and 1,003 healthy control participants (*N*_*PDBP*=_463, *N*_*PPMI*_=141, *N*_*BioFIND*_=76, *N*_*WashU-MDC*_=323; Table 1, Figure 1). Following rigorous QC, we performed DE analyses in each population and integrated the results from all four populations via meta-analysis (Figure 1). In the meta-analysis, we evaluated 2,228 transcripts that had the same direction of effect across all four populations and found 296 transcripts to be DE when comparing Parkinson’s disease to healthy controls, 141 of those downregulated and 155 upregulated (Figure 2A, Supplementary Fig. 1, Supplementary Table 2). While dysregulation of some transcripts, such as *CD55* (*log2FC*=0.07, *p*=6.67×10^−7^) was driven by one of the populations (PPMI in case of *CD55*), six of the 296 DE transcripts, referred hereon after as the six high-confidence transcripts, *NCBP3* (*log2FC*=−0.02, *p*=2.10×10^−9^), *DLD* (*log2FC*=−0.02, *p*=1.96×10^−7^), *RNF169* (*log2FC*=0.04, *p*<2.20×10^−16^), *CCDC57* (*log2FC*=−0.04, *p*=8.84×10^−13^), *MRTFA* (*log2FC*=−2.73, *p*=9.45×10^−12^), and *ZKSCAN8* (*log2FC*=0.02, *p*<2.20×10^−16^; Supplementary Fig. 1, Supplementary Table 2), were nominally significant across all four cohorts and one, *RNF169,* was significant after multiple test correction in each of the four cohorts.

To assess the potential biological relevance of the 296 DE transcripts, we explored the Kyoto Encyclopedia of Genes and Genomes (KEGG) and found that the identified transcripts were significantly enriched in the *nucleocytoplasmic transport pathway* (*p*=4.05×10^−7^; Supplementary Table 3), driven by *NCBP2* (*log2FC*=−0.01, *p*<2×10^−16^). Consistently, Gene Ontology (GO) analysis revealed significant enrichment in *nucleocytoplasmic transport* (*p*=4.57×10^−7^) and *RNA splicing* (*p*=1.81×10^−6^; Figure 2B, Supplementary Table 4) terms. Additionally, to test whether the change in the transcript level is also reflected in the changes in protein level, we accessed publicly available PPMI plasma proteomic data generated with Olink. The 296 DE transcripts corresponded to 279 proteins, six of which were quantified in plasma. We identified two proteins, PROC and SCLY, that were differentially accumulated in Parkinson’s disease patients, and translated from two of the 296 DE transcripts (Supplementary Table 2). Although this does not constitute a significant overlap (*p*=0.15), both proteins exhibited the same direction of effect as their corresponding transcripts in blood. Of the remaining four proteins successfully quantified in plasma, one, SUMF2, exhibited the same direction of change in plasma (effect size=−0.16, *p*=0.09) as its corresponding transcript in blood (*log2FC*=−0.03,*p*=6.40×10^−4^; Supplementary Table 2), though it did not reach significance.

### Genetic ancestry influences the blood transcriptomic landscape

We investigated the influence of ancestry on the identified DE transcripts. We found that 166 of the 296 DE transcripts had the same direction of effect in the non-Hispanic Whites and the Black/African American participants, 16 of which were nominally significant in the Black/African American participants (Figure 2B, Supplementary Table 5). There was no significant difference (*p*=0.80) in effect sizes of the 166 transcripts between the non-Hispanic Whites and the Black/African American participants. Indeed, the effect sizes show strong significant correlation (*ρ*=0.73, *p*=5.17×10^−29^, Supplementary Fig. 2A). None of the six high confidence transcripts that were nominally significant across all four populations in the non-Hispanic White participants were significantly DE in the Black/African American participants, however five of them displayed the same direction of effect in the Black/African American participants, *NCBP3* (effect size=−5.65×10^−3^; *p*=0.78), *DLD* (effect size=−4.05×10^−4^; *p*=0.98), *CCDC57* (effect size=−7.45×10^−3^; *p*=0.71), *MRTFA* (effect size=−0.01, *p*=0.35), and *ZKSCAN8* (effect size=5.10×10^−3^, *p*=0.79; Supplementary Table 5), as observed in the European ancestry participants.

### Mutations exacerbate transcriptomic differences between Parkinson’s disease and healthy controls

To understand how mutations in *LRRK2*, *GBA1*, and *SNCA* could contribute to already identified transcriptional changes we stratified our analyses in Parkinson’s disease participants mutation status, comparing them to our original analysis which included all Parkinson’s disease cases, regardless of mutation status, to maximize statistical power and gain a comprehensive understanding of the transcriptomic landscape in Parkinson’s disease. We found a significant overlap between the number of transcripts that had the same direction of effect and were DE in both the meta-analysis and mutation carriers, *LRRK2*^+^ (203/296, *p*=4.47×10^−92^) and *GBA1*^+^ (178/296, *p*=4.54×10^−95^) carriers compared to *SNCA*^+^ carriers (125/296, *p*=3.21×10^−54^; Figure 2B, Supplementary Table 5). Overall, we observed a strong correlation between effect sizes of DE transcripts in the meta-analysis and *LRRK2* (*ρ*=0.86, *p*=3.57×10^−88^), *GBA1* (*ρ*=0.87, *p*=9.60×10^−92^) and *SNCA* (*ρ*=0.80, *p*=7.23×10^−68^), as well as idiopathic participants (*ρ*=0.85, *p*=6.83×10^−84^, Supplementary Fig. 2B–E). When we focused on the six high confidence transcripts, we observed that for all six the direction of effect in idiopathic participants, as well as mutation carriers, remained the same as in the meta-analysis. Further, two of the six, *RNF169* and *CCDC57*, were DE in both the mutation carriers and idiopathic participants, but with a stronger association and larger effect sizes in mutation carriers (*LRRK2*^+^: *RNF169*, *log2FC*=0.12, *p*=1.46×10^−14^; *CCDC57*, *log2FC*=−0.13, *p*=3.49×10^−8^; *GBA1*^+^: *RNF169*, *log2FC*=0.14, *p*=3.44×10^−12^; *CCDC57*, *log2FC*=−0.22, *p*=3.44×10^−10^; *SNCA*^+^: *RNF169*, *log2FC*=0.19, *p*=4.68×10^−10^; *CCDC57*, *log2FC*=−0.15, *p*=5.73×10^−3^; Supplementary Table 5). Conversely, *DLD* was DE in the mutation carriers (*LRRK2*^+^: *log2FC*=−0.10, *p*=7.65×10^−7^; *GBA1*^+^: *log2FC*=−0.07, *p*=0.03; *SNCA*^+^: *log2FC*=−0.16, *p*=1.24×10^−4^), and not in the idiopathic participants (*log2FC* =−0.02, *p*=0.10; Supplementary Table 5). The expression patterns of the remaining three transcripts showed additional heterogeneity, with *NCBP3* being DE in *LRRK2*^+^ (*log2FC*=−0.03, *p*=7.38×10^−3^) and *SNCA*^+^ (*log2FC*=−0.07, *p*=0.02) carriers, *MRTFA* in idiopathic participants (*log2FC*=−0.03, *p*=0.03) and *LRRK2*^+^ carriers (*log2FC*=−0.06, *p*=1.89×10^−3^), while *ZKSCAN8* was exclusively DE in *SNCA*^+^ carriers (*log2FC*=0.07, *p*=0.01; Supplementary Table 5).

### Transcriptomic perturbations can be detected prior to symptom onset

We next examined whether individuals at risk of developing Parkinson’s disease displayed similar transcriptomic patterns to those observed in symptomatic participants. At risk individuals included carriers of known Parkinson’s disease-related mutations (*LRRK2*^+^, *GBA1*^+^, and *SNCA*^+^), as well as individuals exhibiting Parkinson’s disease-associated syndromes like RBD or hyposmia, none of whom had symptoms of Parkinson’s disease. This included 159 *LRRK2*^+^ and 59 *GBA1*^+^ mutation carriers, as well as 18 participants with RBD and 27 participants with hyposmia from the PPMI cohort. Given that only six *SNCA*^+^ carriers were available, analyses of this group of at-risk participants were not conducted.

We found a significant enrichment in the transcripts that were already dysregulated in *LRRK2*^+^ at-risk participants (193/296, *p*=7.22×10^−92^). Similarly, we observed dysregulation of the DE transcripts in *GBA1*^+^ (166/296, *p*=3.97×10^−72^) at-risk participants, as well as in participants with RBD (126/296, *p*=3.41×10^−53^) and hyposmia (80/296, *p*=9.57×10^−35^) (Figure 2C, Supplementary Table 5). We found that the effect sizes of DE transcripts were strongly correlated between the meta-analysis and all groups of at-risk participants (*LRRK2*^+^: *ρ*=0.81, *p*=3.94×10^−70^; *GBA1*^+^: *ρ*=0.76, *p*=2.64×10^−56^; RBD: *ρ*=0.75, *p*=2.46×10^−54^; HYP: *ρ*=0.70, *p*=1.16×10^−44^, Supplementary Fig. 2F–I). Consistent with our findings in symptomatic participants, we observed that *DLD*, *RNF169*, and *CCDC57* differentially accumulated in *LRRK2*^+^ (*DLD*, *log2FC*=−0.06,=4.20×10^−4^; *RNF169*, *log2FC*=0.09, *p*=6.34×10^−11^; *CCDC57*, *log2FC*=−017, *p*=6.37×10^−14^) and *GBA1*^+^ (*DLD*, *log2FC*=−0.06, *p*=4.49×10^−3^; *RNF169*, *log2FC*=0.08, *p*=3.01×10^−6^; *CCDC57*, *log2FC*=−0.20, *p*=9.06×10^−14^) carriers compared to healthy controls. *MRTFA*, too, was DE in LRRK2^+^ (*log2FC*=−0.06, *p*=1.58×10^−3^) and, unlike in the symptomatic participants, in *GBA1*^+^ (*log2FC*=−0.09, *p*=7.71×10^−5^; Supplementary Table 5) at-risk participants. In contrast, *NCBP3* was DE in *LRRK2*^+^ (*log2FC*=−0.03, *p*=0.04), and not in *GBA1*^+^, while *ZKSCAN8* was DE in *GBA1*^+^ (*log2FC*=0.04, *p*=0.01) and not in *LRRK2*^+^. Moreover, we identified three of the six high confidence transcripts, *RNF169* (*log2FC*=0.11, *p*=2.11×10^−6^), *MRTFA* (*log2FC*=−0.12, *p*=1.37×10^−3^), and *DLD* (*log2FC*=−0.13, *p*=8.24×10^−5^), to be DE in participants with RBD, and two of the six, *RNF169* (*log2FC*=0.06, *p*=0.03) and *CCDC57* (*log2FC*=−0.17, *p*=6.77×10^−3^; Supplementary Table 5), to be DE in participants with hyposmia.

### Transcript expression correlates with symptom severity and Parkinson’s disease medication

To assess the clinical significance of our findings, we explored whether the expression levels of DE transcripts related to severity of motor manifestations, captured in UPDRS-III scores, or dementia, conveyed through MoCA scores. These analyses were carried out in PDBP, PPMI and BioFIND cohorts and integrated via meta-analysis. Information on symptom severity was not available for the WashU-MDC cohort. We found that 123 of the 296 DE transcripts associated significantly with UPDRS-III (Supplementary Table 6), while 106 DE transcripts had a significant association with MoCA (Supplementary Table 6). Of the 106 DE transcripts correlated with MoCA scores, 105 also related to UPDRS-III, such as *CD55* (*P*_*UPDRS-III*_=5.11×10^−12^, *P*_*MoCA*_=4.44×10^−11^), or one of the six high confidence transcripts, *RNF169* (*P*_*UPDRS-III*_=1.13×10^−12^, *P*_*MoCA*_=3.21×10^−11^; Supplementary Table 6). Next, we investigated if the expression of the identified transcripts was affected by medication usage. We observed 89 of the 296 identified transcripts to be significantly associated with levodopa dosage (Supplementary Table 7). Further, we found that expression of three of the six high confidence transcripts, *RNF169* (*log2FC*=2.78×10^−5^, *p*=0.03), *NCBP3* (*log2FC*=−2.31×10^−5^, *p*=2.86×10^−7^) and *MRTFA* (*log2FC*=−4.36 ×10^−5^, *p*=1.36×10^−11^; Supplementary Table 7), associated with medication. Moreover, 34 transcripts affiliated with medication usage also correlated with UPDRS-III scores, while 30 of the 89 medication affiliated transcripts correlated with MoCA scores. We performed mediation analyses to determine whether there was a causal relationship between transcript levels and medication efficacy. We found that two transcripts, *NUP43* (estimate=−4.86×10^−4^; *p*=0.02) and *MAML3* (estimate=−5.67×10^−4^; *p*=0.04), had significant average causal mediation effects.

### Whole blood transcriptomic patterns capture transcriptomic changes in brain

We integrated our findings with an in-house brain RNAseq dataset to identify whether transcriptomic changes in whole blood reflect pathologic changes in the brain. Differentially expressed brain transcripts significantly overlapped with those we identified in whole blood (80/296, *p*=3.60×10^−19^). Of those, 54 transcripts exhibited the same direction of effect in the two tissues, including *DLD* (*log2FC*=−0.29, *p*=0.03; Supplementary Table 2). KEGG pathway enrichment analysis revealed that the 80 transcripts were nominally enriched in PPAR signaling pathway (*p*=0.02, Supplementary Table 3), as well as several immunity-related pathways.

To test whether any of the DE transcripts correspond to proteins differentially accumulated in CSF we accessed publicly available PPMI CSF proteomic data generated with Somalogic. We identified nine (CD55, DLD, MGAT4B, MTPAP, NPLOC4, PDGFD, PLXNC1, POR, PSMD5) differentially accumulated proteins that were translated from nine of the 296 DE transcripts (Supplementary Table 2). While the overlap with transcriptomic changes was not significant (*p*=0.32), six of these proteins exhibited the same direction of effect as their corresponding transcripts in blood.

### Differentially expressed transcripts in blood originate from Parkinson’s disease-associated loci

Next, we inspected if any of the 296 DE transcripts were transcribed from Parkinson’s disease-risk loci. Our analysis revealed 28 transcripts significantly (*p*=1.82×10^−29^) overlapping 16 previously reported Parkinson’s disease GWAS loci^39^ (Supplementary Table 8). Nearly all loci overlapped either one (nine loci) or two (six loci) transcripts, while one locus overlapped with five DE transcripts. Eight of the overlapping transcripts (novel transcript ENSG00000280287, *AP4E1*, *BRD7*, *EBF4*, *GABPB1*, *NTRK1*, *PJA1* and *TRAF3IP2-AS1*) were also DE in the brain. Of the 26 transcripts, one was concordant with the genes nominated by Nalls *et al*.^46^ or Kim *et al*.^47^, whereas 25 would nominate a different gene. Our analyses suggest that for the region chr22:41434158 the gene driving the association is *MRTFA* instead of either of the eight putative genes nominated (*ZC3H7B*, *POLR3H*, *CSDC2*, *PMM1*, *RANGAP1*, *MEI1*, *L3MBTL2*, *SLC25A17*). For some regions such as chr3:48711556 or chr12:132487182 several transcripts are DE, which suggests that several genes are driving the association or that the region is overall dysregulated (Supplementary Table 8). Further, we found that two DE transcripts, *NAA10* and *PJA1*, overlapped Parkinson’s disease-associated loci on the X chromosome,^40^ which constituted a significant (*p*=4.89×10^−4^) overlap. Additionally, we browsed the Parkinson’s disease GWAS Locus Browser^41^ to find if any of the DE transcripts originated from genes that had supporting evidence as Parkinson’s disease-causal genes. We discovered that twelve DE transcripts were produced from genes with an average score of 4.75 (±1.96) (Supplementary Table 8). Moreover, of the twelve transcripts, two, *BRD7* and *TRAF3IP2-AS1*, were also DE in brain (Supplementary Table 2).

We also performed expression quantitative trait locus (eQTL) mapping to identify genomic regions regulating the DE transcripts and to evaluate their potential causal association with Parkinson’s disease. Out of the 296 identified transcripts, 15 were found to have genome-wide significant *cis*-eQTLs (*p*<5.00×10^−8^; Supplementary Table 9, Supplementary Fig. 3). To assess their relationship with Parkinson’s disease, we conducted two-sample MR using summary statistics from the latest Parkinson’s disease GWAs, which revealed 13 regions nominally associated with Parkinson’s disease. Eleven of the 13 regions were already identified by the latest Parkinson’s disease GWAS. For example, for region chr6:32411770 our analysis suggests that *BRD2* (*p*=0.02) is the gene driving the association instead of *HLA-DRB5*. Similarly, for region chr1:155070849 we found *CLK2* (*p*=4.81×10^−5^) and *SCAMP3* (*p*=4.81×10^−5^; Supplementary Table 9) to be the drivers of the association instead of previously nominated *GBAP1*. The transcripts encoded by new regions and significant in the MR analyses (*MRPL42* and *PIGN*) represent additional genes associated with Parkinson’s disease. We also found suggestive signals for regions not identified by previous studies, such as *CD55* (*p*=1.88×10^−3^) in the chr1:208152457 region. None of these associations remained significant after correction for multiple testing.

### Whole blood captures Parkinson’s disease associated transcriptomic signatures even before symptom onset

**Figure 3:**
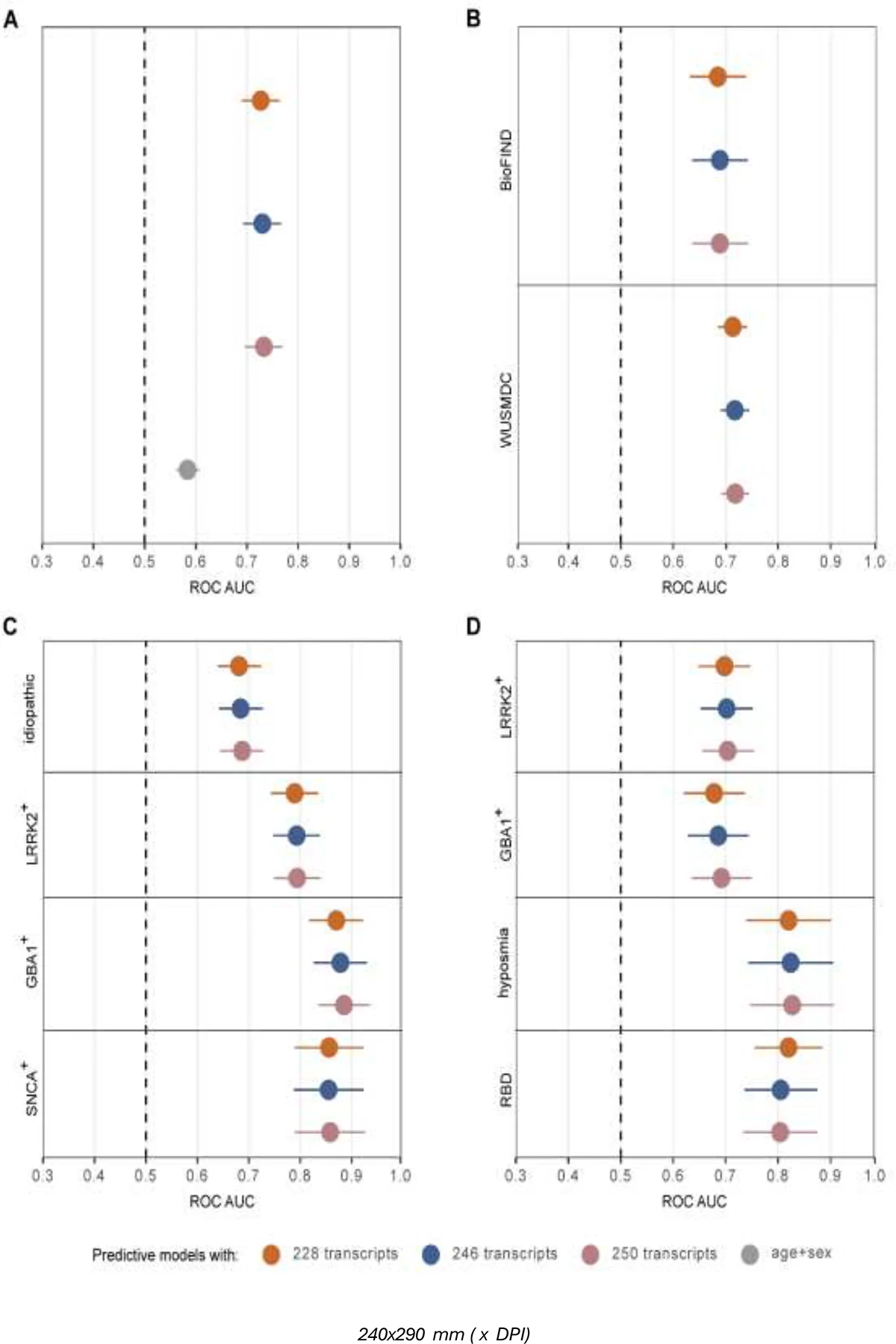
Predictive models accurately differentiate PD from healthy controls. Whisker plots showing predictive model performance in the **(A)** testing population, **(B)** validation populations, **(C)** symptomatic participants stratified by mutation status, and **(D)** at-risk participants.

We explored if the DE transcripts can distinguish between Parkinson’s disease and healthy control participants. The four independent RNAseq datasets were used to train (PDBP), test (PPMI) and validate (WashU-MDC and BioFIND) predictive models, following the previously developed approach.^44^ We evaluated 100 predictive models equating to 100 KLD value thresholds (between 0.01 and 1) and selected the three models that had best balanced AUC-ROC values and number of transcripts (Supplementary Fig. 4). The selected models contained 228, 246 and 250 transcripts and achieved AUCs of 0.727, 0.730 and 0.733, respectively, in the testing dataset (Figure 3A, Supplementary Table 10). We found that the predictive models performed better than age and sex alone which achieved an AUC of 0.584 (0.561–0.608), in predicting Parkinson’s disease. Further, the addition of age and sex information to the predictive models lead to a marginal improvement in their performance, increasing the AUC values to 0.736, 0.742 and 0.742, for models containing 228, 246 and 250 transcripts, respectively. Similarly, the exclusion of medication associated transcripts did not improve model performance, achieving AUC of 0.708 (0.669–0.747), lower than any of our three models (Supplementary Fig. 5). The models were validated, exhibiting similar AUC values in WashU-MDC (*AUC*_*228*_=0.716, *AUC*_*246*_=0.721 and *AUC*_*250*_=0.722) and BioFIND (*AUC*_*228*_=0.680, *AUC*_*246*_=0.685 and *AUC*_*250*_=0.685; Figure 3B, Supplementary Table 10) populations. We further explored if genetic background and ancestry affected predictive power of the selected models. We observed that all three models had higher predictive power in symptomatic carriers of *LRRK2* mutations (*AUC*_*228*_=0.789, *AUC*_*246*_=0.793 and *AUC*_*250*_=0.794), *GBA1* (*AUC*_*228*_=0.870, *AUC*_*246*_=0.878 and *AUC*_*250*_=0.885) and *SNCA* mutations (*AUC*_*228*_=0.856, *AUC*_*246*_=0.855 and *AUC*_*250*_=0.858), compared to idiopathic participants (*AUC*_*228*_=0.681, *AUC*_*246*_=0.684 and *AUC*_*250*_=0.687; Figure 3C, Supplementary Table 10). On the other hand, when testing in African-American participants, the models performed comparable to validation in Washu-MDC and BioFIND, achieving AUCs of 0.694, 0.726 and 0.720 for models containing 228, 246 and 250 transcripts, respectively (Supplementary Fig. 6, Supplementary Table 10). Finally, we inspected whether the designed models could differentiate between at-risk and healthy control participants. The model performance in at-risk *LRKK2*^+^ (*AUC*_*228*_=0.698, *AUC*_*246*_=0.702 and *AUC*_*250*_=0.704) and *GBA*^+^ (*AUC*_*228*_=0.678, *AUC*_*246*_=0.686 and *AUC*_*250*_=0.692) mutation carriers was similar to that in idiopathic Parkinson’s disease participants, while they achieved higher AUC values in participants with hyposmia (*AUC*_*228*_=0.820, *AUC*_*246*_=0.824 and *AUC*_*250*_=0.827) and RBD (*AUC*_*228*_=0.820, *AUC*_*246*_=0.805 and *AUC*_*250*_=0.804; Figure 3D, Supplementary Table 10).

## Discussion

In the current study, we leveraged four large-scale whole-blood RNA-seq datasets to identify transcripts that differentially accumulated in participants with Parkinson’s disease. To our knowledge, this represents the largest and most comprehensive Parkinson’s disease transcriptomic analysis to date,^11,13,48–51^ encompassing data from 2,628 participants across the PDBP, PPMI, BioFIND, and WashU-MDC, while also addressing distinct Parkinson’s disease subpopulations and multi-omic data integration. We identified 296 transcripts that were DE in Parkinson’s disease participants, many of which are implicated in pathways associated with neurodegeneration, such as nucleocytoplasmic transport.^52–54^ Furthermore, our analysis suggested that expression differences are influenced by ethnic background, highlighting the need for multi-ancestry approaches in Parkinson’s disease research. Finally, we leveraged our findings to develop three scalable predictive models that achieved AUCs of 0.727–0.733 in the testing population. Unlike previous studies^14,51^, we used two independent cohorts to validate our models, showing that they maintain similar levels of accuracy in the validation populations. While we show that reliable, replicable predictive models can be based on transcriptomic data alone, like the previous study^14^ we suggest that the performance of the models improves by inclusion of other, non-transcriptomic features, such as genetic and clinical information, as we observed in our models with Parkinson’s disease-associated mutations, though these were also limited in power due to a limited number of mutation carriers. Similarly, the emerging biomarkers under development for Parkinson’s disease diagnosis, such as α-synuclein seed amplification assay (α-syn SAA) and advanced neuroimaging, have had varied levels of success. For example, albeit some α-syn SAA protocols achieved reported 80.39% sensitivity and 92.43% specificity in serum samples, whole blood contains proteins and lipids that can interfere with seed amplification, which leads to lower sensitivity.^55,56^ Advanced neuroimaging performs well at differentiating between Parkinson’s disease and healthy controls, with studies reporting perfect sensitivity (100%), but the specificity seems to be highly varied and dependent on the imaging technology, being as low as 53% in some reports.^57,58^ Further, cost, accessibility, and radiation exposure present substantial practical constraints to neuroimaging approaches. Altogether, we propose that transcriptomic features, combined with other clinical information, pose a promising low cost minimally invasive biomarkers.

We found that 28 of the 296 identified transcripts originated from known Parkinson’s disease-associated loci.^40,46,47^ Eight of these transcripts were also DE in brain, adding evidence to their involvement in Parkinson’s disease, while the blood-specific transcripts could be contributing to other, non-neuronal, aspects of the disease pathophysiology. Notably, two of the 28 transcripts, *STAT3* and *QRICH1*, scored an eight in the Parkinson’s disease GWAS locus browser,^41^ comparable to the well-established Parkinson’s disease gene *GBA1*, which has a score of nine. *STAT3* has been proposed as a therapeutic candidate for Parkinson’s disease due to its neuroprotective effects. Overexpression of *STAT3* has been shown to protect dopaminergic neurons from degeneration in a Parkinson’s disease rat model.^59^ Despite not being significant in the MR analyses, we found significant correlation between *STAT3* and clinical measures of motor signs (UPDRS-III) and cognitive decline (MoCA), providing further support for the involvement of *STAT3*. In contrast, *QRICH1,* while not previously related to Parkinson’s disease, has been associated with polymicrogyria, a neurodevelopmental disorder characterized by cognitive deficits and motor abnormalities.^60^ Additionally, two of the 28 transcripts, *NAA10* and *PJA1*, originate from loci on the X chromosome and have established roles in neurodegeneration. Given the location of these genes, further studies might investigate if they could explain, fully or partially, the higher prevalence of Parkinson’s disease in males via dosage effect. As the catalytic subunit of the NatA complex, NAA10 is responsible for N-terminal acetylation of approximately 40–50% of all human proteins.^61–63^ This process regulates amyloid β-protein generation, modulates the stabilization of Sup35 amyloid formation, and prevents aggregation of Htt, further highlighting its importance in neurodegenerative diseases such as Parkinson’s, Alzheimer’s, and Huntington’s diseases.^64–69^ PJA1 facilitates the degradation of ataxin-3 and huntingtin polyglutamine proteins and can suppress the aggregation of FUS, SOD1, and α-synuclein in neuronal cell models.^70,71^ Together, these findings suggest that *PJA1* plays a broad protective role in suppressing protein aggregation and mitigating neurodegenerative processes. Additionally, MR analyses revealed several genes nominally associated with Parkinson’s disease and nominated novel Parkinson’s disease-causal genes. For example, we found that instead of *HLA-DRB5, BRD2* seems to be driving the association in the region chr6:32411770. Recent reports found that BRD2 expression was induced by α-synuclein, bringing upon neuroinflammation typical of Parkinson’s disease,^72^ further supporting our findings.

Transcriptional changes in blood were mirrored by altered protein accumulation in plasma. Namely, PROC and SCLY, which are translated from the identified transcripts, showed dysregulation in plasma. Elevated PROC levels have been linked to a decreased risk of dementia,^73^ while SCLY mediates selenoprotein turnover, which is disrupted in Parkinson’s disease.^74–76^ Further, we found that 80 of the 296 DE transcripts in blood were also DE in brain, supporting the notion of blood-brain barrier leakage in Parkinson’s disease.^77^ These transcripts were also enriched in the PPAR signaling pathway, a potential therapeutic target with neuroprotective effects in Parkinson’s disease.^78,79^ The downregulation of *PSMD5*, a proteolysosomal gene, which was dysregulated on both transcript level in blood and protein level in CSF, has been associated with Parkinson’s disease pathology in myeloid cells, where it plays a role in regulating protein degradation and maintaining cellular homeostasis.^80^ In fact, protein products of nine of the 296 DE transcripts (*CD55, DLD, MGAT4B, MTPAP, NPLOC4, PDGFD, PLXNC1, POR,* and *PSMD5*) differentially accumulated in CSF, six with the same direction of effect in blood and CSF, further validating the relevance of our blood findings to Parkinson’s disease pathobiology and nominating these proteins for further investigation as biomarkers.

In addition to being DE in blood transcriptomic and CSF proteomic data, we identified a strong cis eQTL for *CD55* that was suggestive in the MR, pointing to its possible role in the pathobiology of Parkinson’s disease. On top of that, we found that *CD55* expression levels correlated significantly with UPDRS-III and MoCA scores. Finally, *CD55* was included in all three predictive models. Upregulation of CD55 has been shown to inhibit complement-mediated synaptic phagocytosis in a Parkinson’s disease mouse model.^81^ CD55, also known as decay-accelerating factor (DAF), inhibits the complement cascade, which has been implicated in the pathogenesis of Parkinson’s disease through its association with α-synuclein and Lewy bodies. *CD55* was shown to be dysregulated in Parkinson’s disease, but not AD, further underscoring its potential role in Parkinson’s disease-specific immune dysregulation.^82^ Furthermore, research by Wang *et al*. highlights a broader role for CD55 in modulating the complement system, particularly through its inhibition of the C1q-dependent complement pathway. This pathway is central to microglia-mediated synapse elimination, where CD55 disrupts complement activity and microglial phagocytosis.^83^ Altogether, this suggests that *CD55* has a potential as a marker of disease progression and potential therapeutic target.

Similar to *CD55, BRD7* was found to be DE in whole blood and brain tissue and mapped to a Parkinson’s disease GWAS locus. It was further validated through eQTL mapping despite not being significant in the MR, highlighting its potentially important role. BRD7 is a bromodomain-containing protein (BCP) that has been implicated in the regulation of chromatin and gene expression. It is widely expressed in various brain regions, particularly the medial prefrontal cortex (mPFC), and localizes specifically to neurons. Inactivation of BRD7 in animal models impairs cognitive behavior and reduces synaptic plasticity, particularly in the mPFC.^84^ Additionally, BRD7 plays an anti-inflammatory role by inhibiting the NF-κB signaling pathway, a key pathway involved in neuroinflammation. BRD7 knockout mice have increased inflammation and elevated levels of inflammatory markers,^85^ supporting the idea that BRD7’s normal function helps regulate neuroinflammatory processes. Moreover, recent research has shown that BRD7 complexes with Olig2, a key transcription factor, and interacts with the nucleoporin Seh1 to regulate oligodendrocyte differentiation and myelination in the central nervous system.^86^ This interaction is important for maintaining proper neural function, and myelination defects are common in neurodegenerative diseases, including Parkinson’s disease, where the integrity of dopaminergic neurons is often compromised. These findings suggest BRD7’s central role in neuronal survival, synaptic integrity, and myelination, with potential implications for Parkinson’s disease pathophysiology.

This study also included symptomatic participants with mutations in *LRRK2*, *GBA1* and *SNCA,* as well as asymptomatic individuals at high risk for Parkinson’s disease, such as *LRRK2* and *GBA1* mutation carriers or those with RBD or hyposmia. Notably, several transcripts were dysregulated before symptom onset. Among these, *DLD* was DE in whole blood and showed nominally significant dysregulation across all four subpopulations supporting and broadening previous reports.^87^ On top of that, analyses stratified by mutation carrier status revealed that the association between *DLD* expression and Parkinson’s disease is primarily driven by Parkinson’s disease-related mutations, with further evidence indicating that the expression of *DLD* in at-risk participants is already altered prior to symptom onset. Additionally, *DLD* was not only differentially expressed in whole blood but also exhibited differential accumulation in brain tissue and CSF and was causally linked to Parkinson’s disease through MR analyses. Suppression of DLD has been linked to neurodegenerative disorders, including Alzheimer’s disease, where it protects against Aβ-induced pathology.^88^ Notably, genetic studies have linked the *DLD* locus to an increased risk of late-onset AD,^89^ suggesting a potential genetic vulnerability that could be extended to other neurodegenerative conditions, including Parkinson’s disease. *DLD* is a key subunit of both the alpha-ketoglutarate dehydrogenase and pyruvate dehydrogenase complexes, and reduced activity of these enzymes has been observed in post-mortem brain tissues and fibroblasts from patients with AD and Parkinson’s disease.^90–95^ This points to a possible connection between impaired energy metabolism and neurodegeneration. Given the central role of *DLD* in mitochondrial energy production, its dysregulation may lead to cellular energy deficits, oxidative stress, and neuronal dysfunction—all hallmarks of Parkinson’s disease pathology. Lastly, *DLD* was included in all three predictive models developed here, positioning *DLD* as a promising candidate for future studies as a potential early-stage biomarker or therapeutic target due to its potential causality.

The current study has several limitations. First, the sample size, despite being the largest, is still limited, especially when considering the described subgroups. In other words, despite performing sensitivity analyses to assess the impact that ancestry and genetic background have on transcriptomic landscape, the relatively small sample size of non-European ancestry populations, mutation carriers, and at-risk participants may limit the generalizability of our findings. Further, at-risk group was very heterogenous, consisting of participants with different probabilities of developing Parkinson’s disease. While informative, sub-analyses separating participants by risk factor have limited power and would benefit from more detailed clinical information, including a cumulative risk score, to more accurately stratify at-risk population. Though cleaner genetic background partly explains the superior performance of predictive models in mutation carriers, we recognize that this could also be driven by the small sample size, leading to overfitting. Additionally, lack of detailed clinical and genetic information might have hampered the predictive capabilities of the predictive models. While our analyses suggest that genetic background affects the model performance, detailed mutation carrier information must be confirmed. Similarly, although the predictive models appear to be agnostic of ethnic background, greater multi-ancestry sampling is necessary to confirm these results. Lastly, insufficient detail on medication status (ON or OFF) during UPDRS-III evaluation is a constraint on our mediation analyses, as it poses a possibility that the identified association results from confounding effects of disease severity and compounded treatment exposure, rather than a true causal pathway between medication and symptom severity mediated at the transcriptome level.

Limitations notwithstanding, we have robustly identified Parkinson’s disease-associated transcripts in the largest Parkinson’s disease transcriptomic study to date, proposed novel genes, and biomarker and therapeutic targets. We used multi-omic data integration to demonstrate that whole blood captures signatures of pathobiology. We have also developed scalable, transcript based, predictive models that can differentiate between Parkinson’s disease and healthy controls. Moreover, by incorporating all our observations together, we identified two transcripts, *CD55* and *DLD*, which showed great promise as highly informative early-stage biomarkers of Parkinson’s disease. Altogether, we demonstrated that whole blood can be leveraged for development of minimally invasive biomarkers that could aid in diagnosing Parkinson’s disease in symptomatic and asymptomatic phases.

## Supplementary Material

supplemental data

supplemental data 2

Supplementary material is available at *Brain* online.

## Figures and Tables

**Table 1:** 

	Parkinson’s Progression Markers Initiative (PPMI)		Parkinson’s Disease Biomarkers Program (PDBP)		Washington University in St. Louis Movement Disorder Clinic (WashU-MDC)		Fox Investigation for New Discovery of Biomarkers (BioFIND)	
	Healthy Controls	Parkinson’s Disease	Healthy Controls	Parkinson’s Disease	Healthy Controls	Parkinson’s Disease	Healthy Controls	Parkinson’s Disease
Participants(N)	141	534	463	725	323	260	76	106
MeanAge(IQR)	63.79 (59.00–71.00)	63.87 (57.00–71.00)	62.84 (56.00–71.00)	65.81 (60.00–72.00)	69.25 (63.00–75.00)	69.15 (63.00–76.25)	65.88 (60.75–70.00)	68.17 (64.00–73.00)
Female(N,%)	52 (36.88%)	213 (39.89%)	254 (54.86%)	269 (37.1%)	181 (56.04%)	101 (38.85%)	40 (52.63%)	38 (35.85%)
MeanUPDRS-III(IQR)	1.48 (0.00–2.00)	24.84 (15.00–33.00)	2.12 (0.00–2.00)	25.83 (16.00–33.00)	-	-	-	39.07 (29.25–48.75)
MeanMoCA(IQR)	27.38 (26.00–29.00)	26.36 (25.00–29.00)	26.62 (25.00–28.50)	25.12 (23.00–28.00)	-	-	27.99 (27.00–29.00)	26.85 (26.00–29.00)

## Data Availability

PDBP, PPMI and BioFIND data is available from AMP-PD (https://amp-pd.org/). WashU-MDC data Summary results for all transcripts quantified across all four cohorts can be found in our transcript level browser (pdbloodtranscriptomics.WashU-MDC.edu). All original code has been deposited at GitHub (https://github.com/Ibanez-Lab/BloodLinearRNA-ParkinsonsDisease) and is publicly available as of the date of publication.
