# Supplementary material for: Study of blood linear RNA nominates CD55 and DLD as early-stage biomarkers for Parkinson's disease
Source: Brain. 2026 Jan 13;149(10):3509–23. doi: 10.1093/brain/awag014 (PMC13584185; doi:10.1093/brain/awag014)

A Comprehensive Study of Circulating Blood Linear RNA nominates *CD55* and *DLD* as early-stage biomarkers for Parkinson's Disease

Aleksandra Beric*[1,2], Sarp Sahin*[1,2], Santiago Sanchez[1,2], Zining Yang[1,2], Ravindra Kumar[1,2], Isabel Alfradique-Dunham[3], Jessie Sanford[1,2], Daniel Western[1,2], Bridget Phillips[1,2], John P. Budde[1,2], Joel S. Perlmutter[3,4,5,6,7], Scott A. Norris[3,4], Carlos Cruchaga[1,2,3,8,9,10], Laura Ibanez[1,2,3]

1- Department of Psychiatry, Washington University in Saint Louis School of Medicine
2- NeuroGenomics and Informatics Center, Washington University in Saint Louis School of Medicine
3- Department of Neurology, Washington University in Saint Louis School of Medicine
4- Department of Radiology, Washington University in Saint Louis School of Medicine
5- Department of Neuroscience, Washington University in Saint Louis School of Medicine
6- Program of Physical Therapy, Washington University in Saint Louis School of Medicine
7- Department of Occupational Therapy, Washington University in Saint Louis School of Medicine
8- Hope Center for Neurological Disorders, Washington University in Saint Louis School of Medicine
9- The Charles F. and Joanne Knight Alzheimer Disease Research Center, Washington University in Saint Louis
10- Department of Genetics, Washington University in Saint Louis School of Medicine

* - denotes co-first authors

Correspondence:
Laura Ibanez, PhD
Washington University in Saint Louis School of Medicine
4444 Forest Park Ave
Campus Box 8134
Saint Louis, MO 63110
Tel: 314-273-3641
ibanezl@wustl.edu

Supplementary Figure 1. Differential expression analyses. Volcano plots showing results of differential expression analyses in A. PDBP (maroon), B. PPMI (dark blue), C. BioFIND (light yellow) and 4. WashU-MDC (dark rosy-brown)

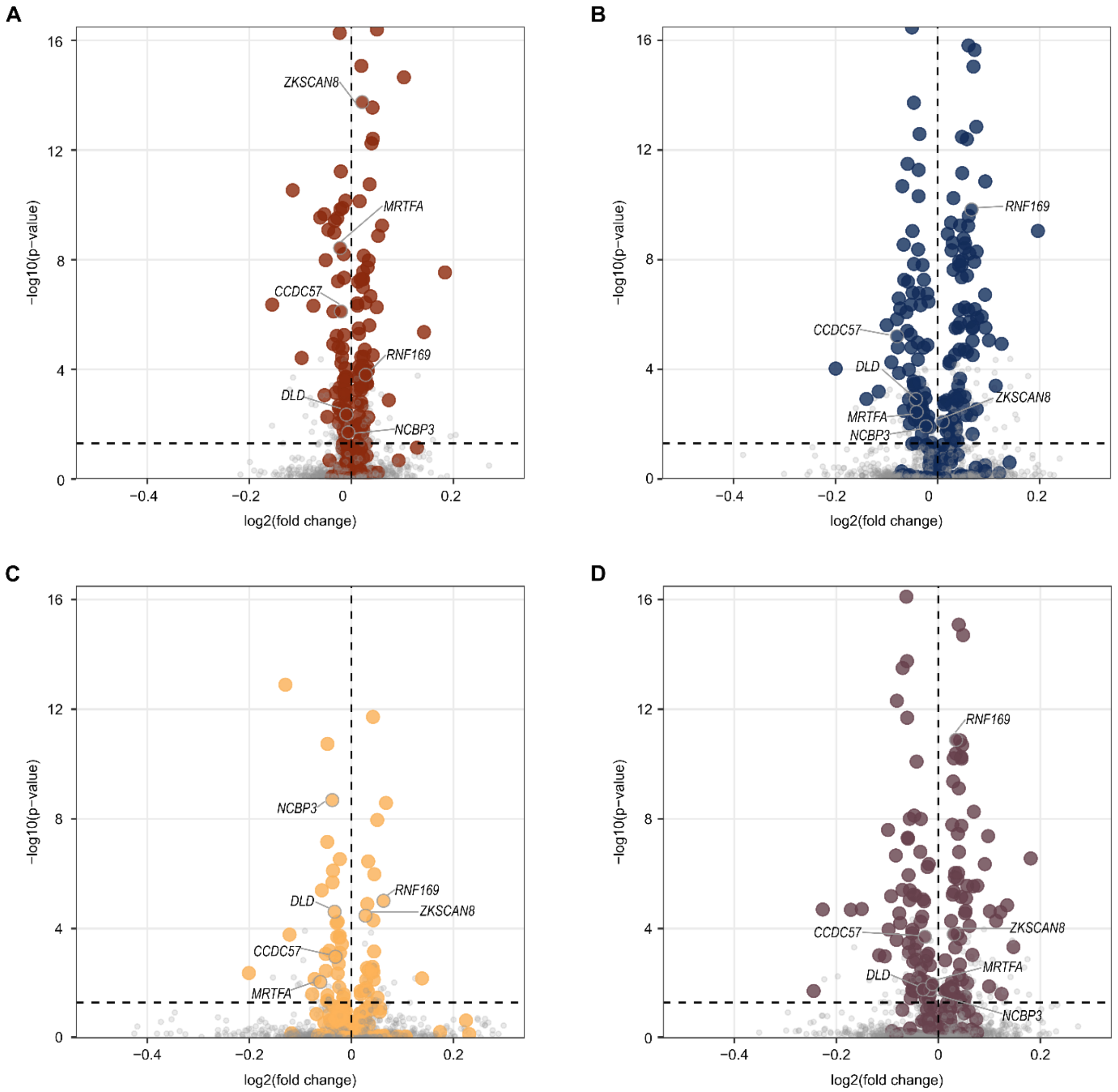

Supplementary Figure 2. Correlation.

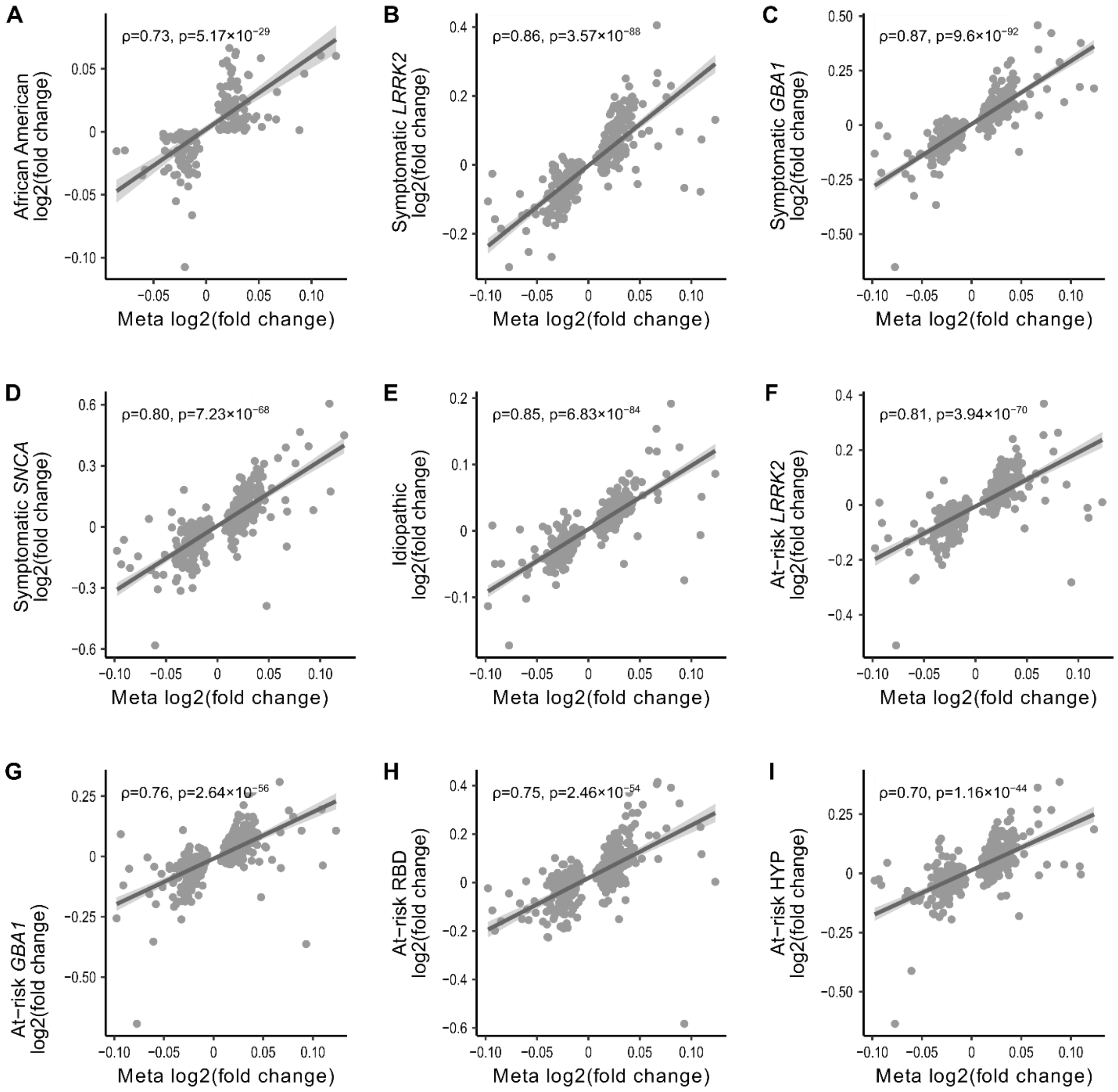

Supplementary Figure 3. Local Manhattan plots.

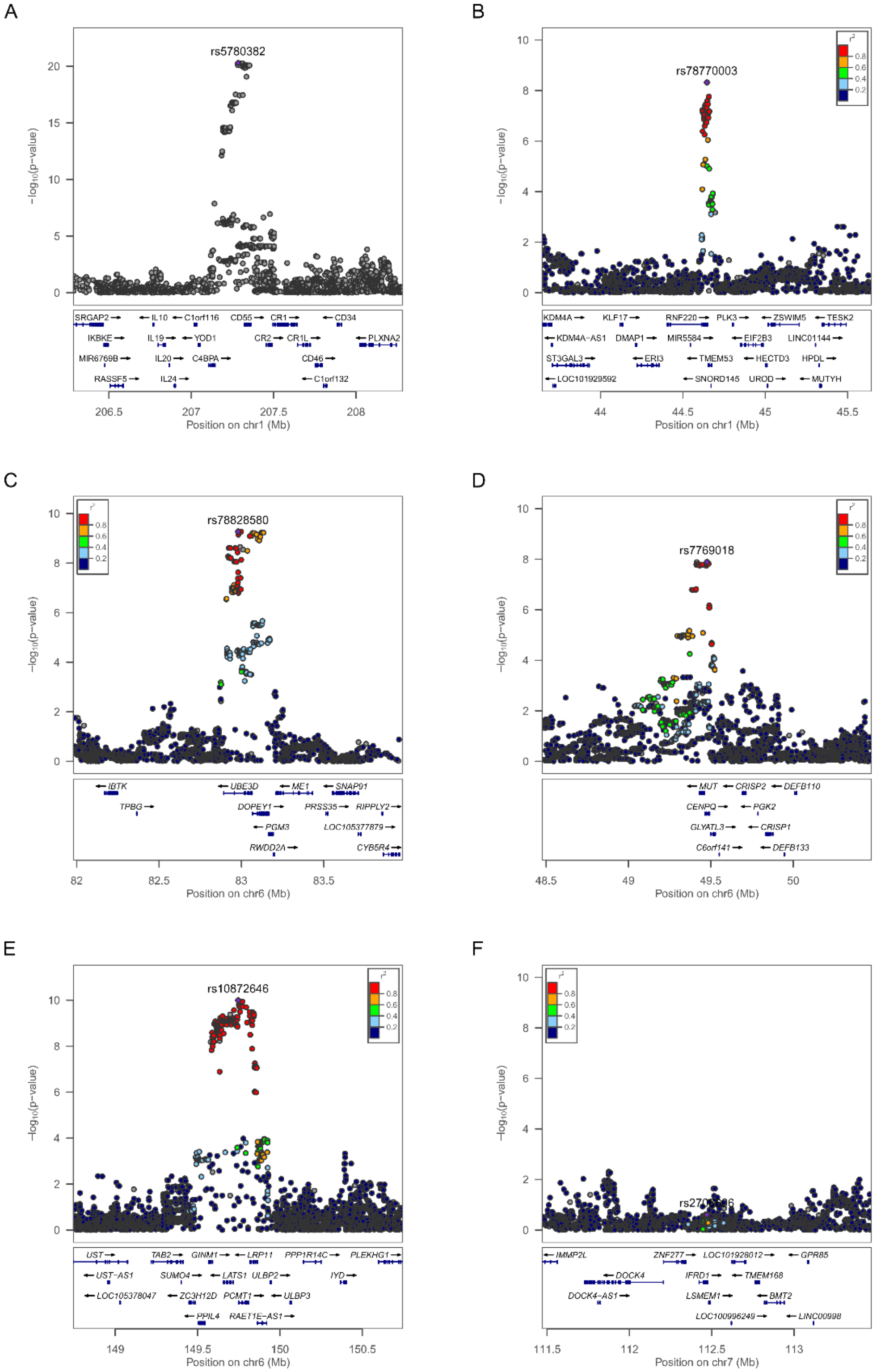

G

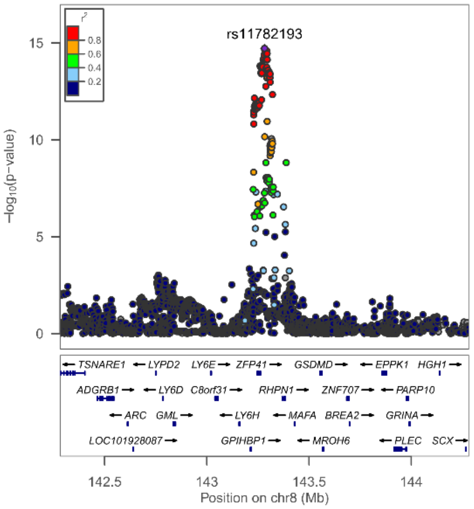
rs11782193
−log10(p−value)
Position on chr8 (Mb)
TSNARE1
LYPD2
LY6E
ZFP41
GSDMD
EPPK1
HGH1
ADGRB1
LY6D
C8orf31
RHPN1
ZNF707
PARP10
ARC
GML
LY6H
MAFA
BREA2
GRINA
LOC101928087
GPIHBP1
MROH6
PLEC
SCX

H

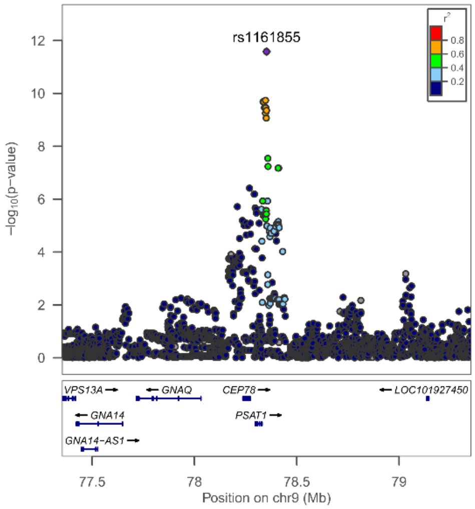
rs1161855
−log10(p−value)
Position on chr9 (Mb)
VPS13A
GNAQ
CEP78
LOC101927450
GNA14
PSAT1
GNA14−AS1

I

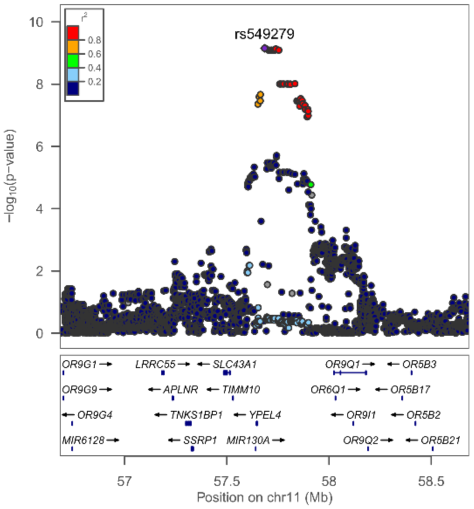
rs549279
−log10(p−value)
Position on chr11 (Mb)
OR9G1
LRRC55
SLC43A1
OR9Q1
OR5B3
OR9G9
APLNR
TIMM10
OR6Q1
OR5B17
OR9G4
TNKS1BP1
YPEL4
OR9I1
OR5B2
MIR6128
SSRP1
MIR130A
OR9Q2
OR5B21

J

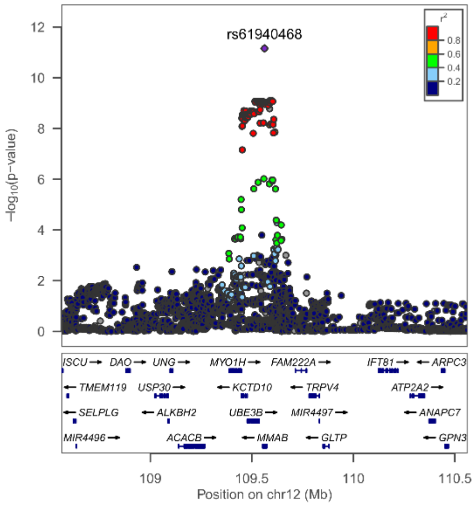
rs61940468
−log10(p−value)
Position on chr12 (Mb)
ISCU
DAO
UNG
MYO1H
FAM222A
IFT81
ARPC3
TMEM119
USP30
KCTD10
TRPV4
ATP2A2
SELPLG
ALKBH2
UBE3B
MIR4497
ANAPC7
MIR4496
ACACB
MMAB
GLTP
GPN3

K

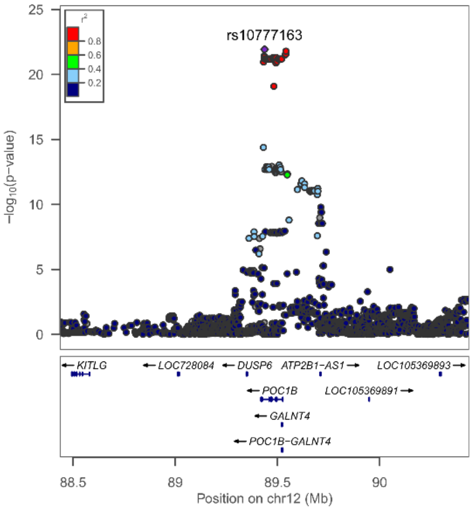
rs10777163
−log10(p−value)
Position on chr12 (Mb)
KITLG
LOC728084
DUSP6
ATP2B1−AS1
LOC105369893
POC1B
LOC105369891
GALNT4
POC1B−GALNT4

L

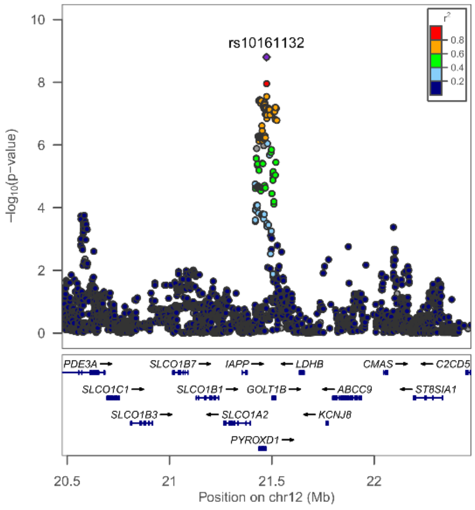
rs10161132
−log10(p−value)
Position on chr12 (Mb)
PDE3A
SLCO1B7
IAPP
LDHB
CMAS
C2CD5
SLCO1C1
SLCO1B1
GOLT1B
ABCC9
ST8SIA1
SLCO1B3
SLCO1A2
KCNJ8
PYROXD1

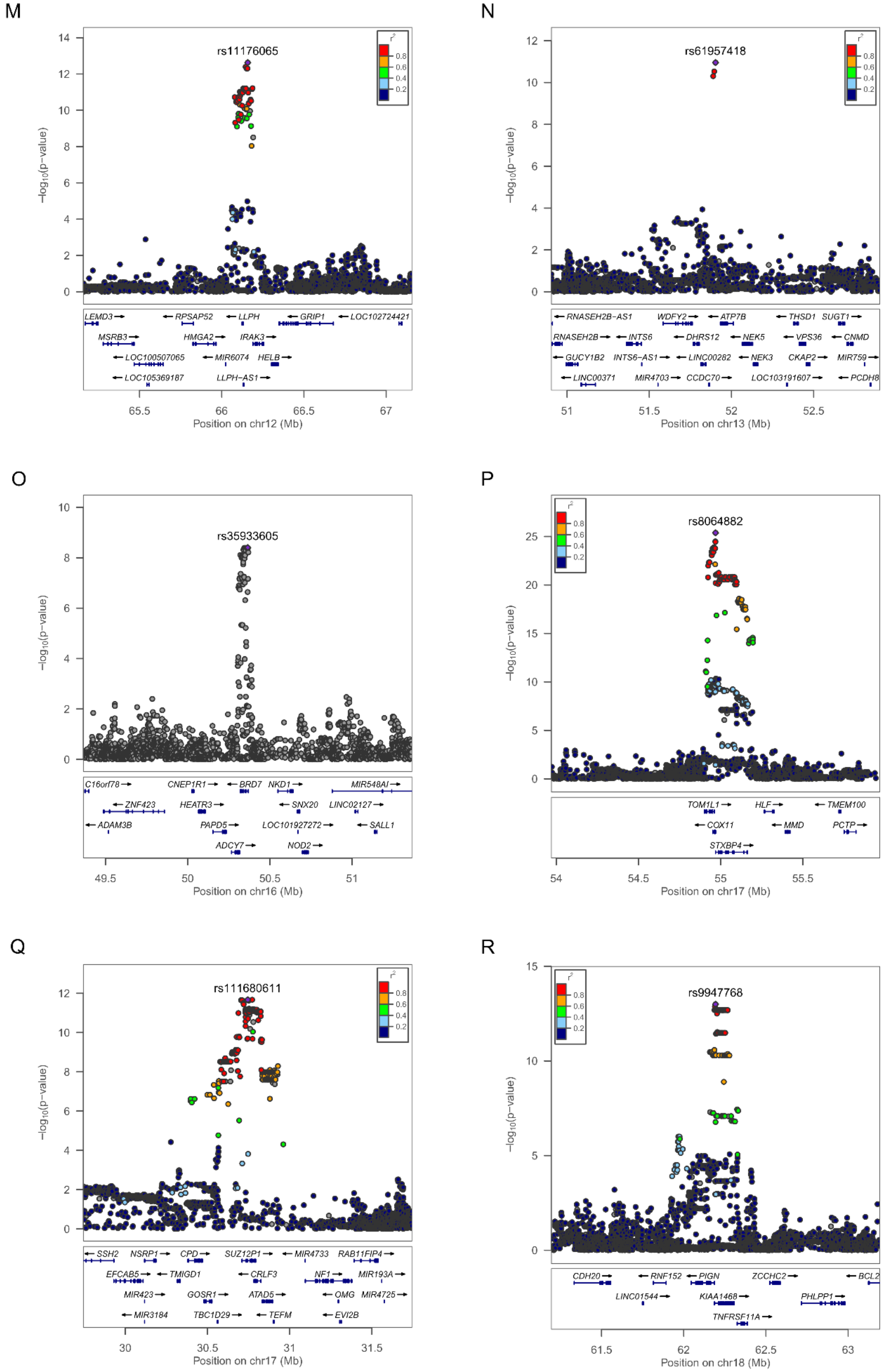
M
rs11176065
−log10(p−value)
Position on chr12 (Mb)
LEMD3
RPSAP52
LLPH
GRIP1
LOC102724421
MSRB3
HMGA2
IRAK3
LOC100507065
MIR6074
HELB
LOC105369187
LLPH-AS1
N
rs61957418
−log10(p−value)
Position on chr13 (Mb)
RNASEH2B-AS1
WDFY2
ATP7B
THSD1
SUGT1
RNASEH2B
INTS6
DHRS12
NEK5
VPS36
CNMD
GUCY1B2
INTS6-AS1
LINC00282
NEK3
CKAP2
MIR759
LINC00371
MIR4703
CCDC70
LOC103191607
PCDH8
O
rs35933605
−log10(p−value)
Position on chr16 (Mb)
C16orf78
CNEP1R1
BRD7
NKD1
MIR548AI
ZNF423
HEATR3
SNX20
LINC02127
ADAM3B
PAPD5
LOC101927272
SALL1
ADCY7
NOD2
P
rs8064882
−log10(p−value)
Position on chr17 (Mb)
TOM1L1
HLF
TMEM100
COX11
MMD
PCTP
STXBP4
Q
rs111680611
−log10(p−value)
Position on chr17 (Mb)
SSH2
NSRP1
CPD
SUZ12P1
MIR4733
RAB11FIP4
EFCAB5
TMIGD1
CRLF3
NF1
MIR193A
MIR423
GOSR1
ATAD5
OMG
MIR4725
MIR3184
TBC1D29
TEFM
EVI2B
R
rs9947768
−log10(p−value)
Position on chr18 (Mb)
CDH20
RNF152
PIGN
ZCCHC2
BCL2
LINC01544
KIAA1468
PHLPP1
TNFRSF11A

Supplementary Figure 4. Feature Selection. Model performance in the training population for increasing KLD cutoff values. The Y-axis represents the AUC of each model, and the X-axis represents the KLD value used as threshold, with models selected for further analyses highlighted in orange.

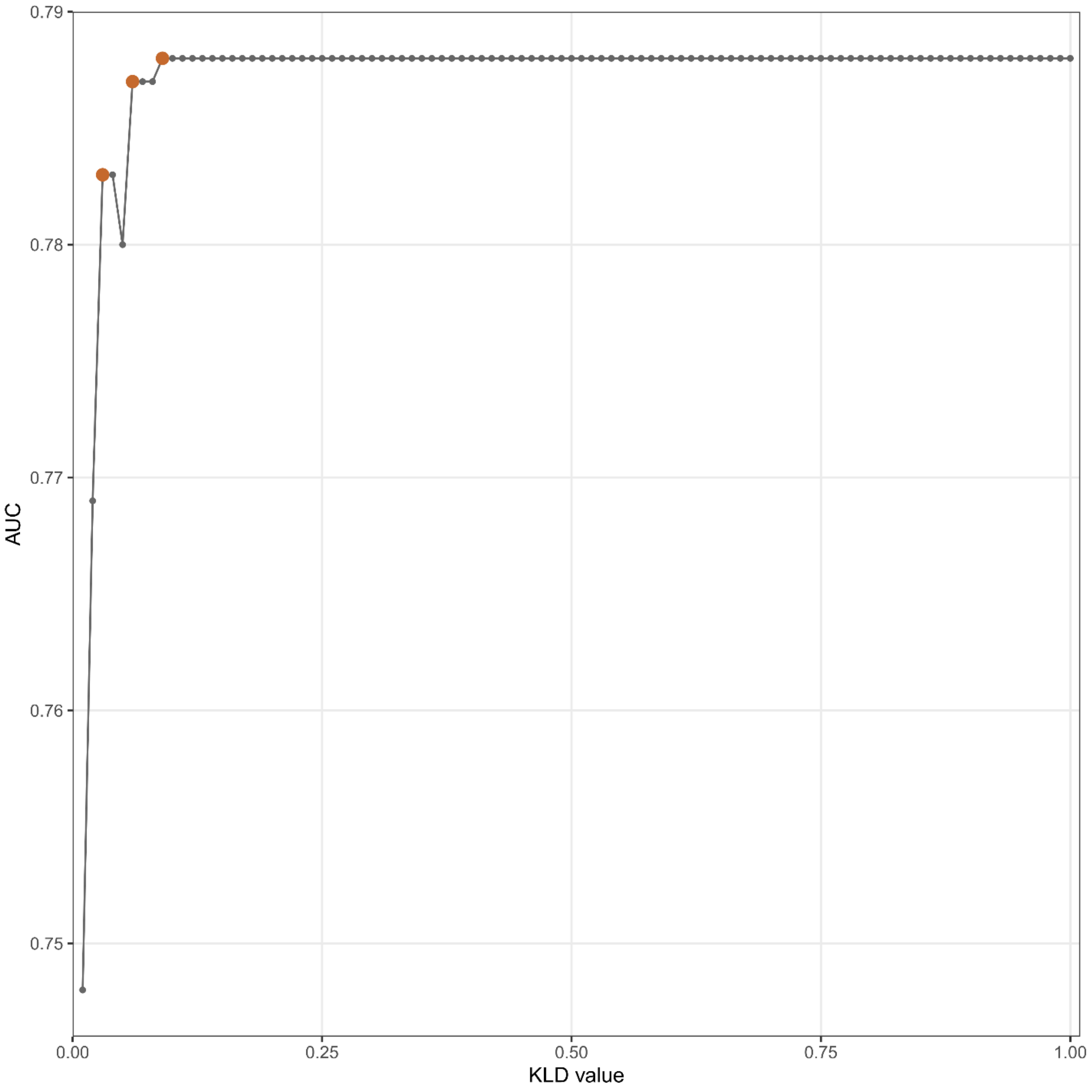

Supplementary Figure 5. Performance of the model excluding medication associated transcripts

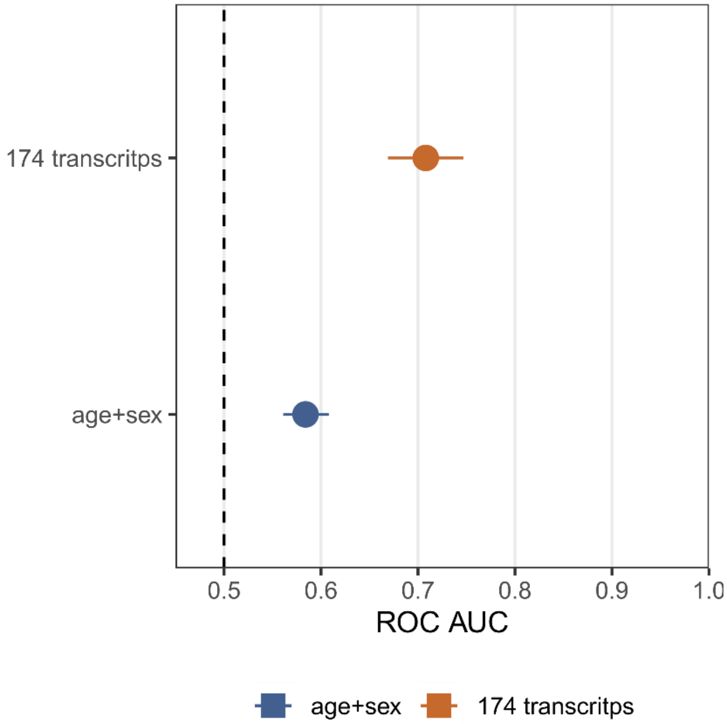

Supplementary Figure 6. Model performance in African-American participants

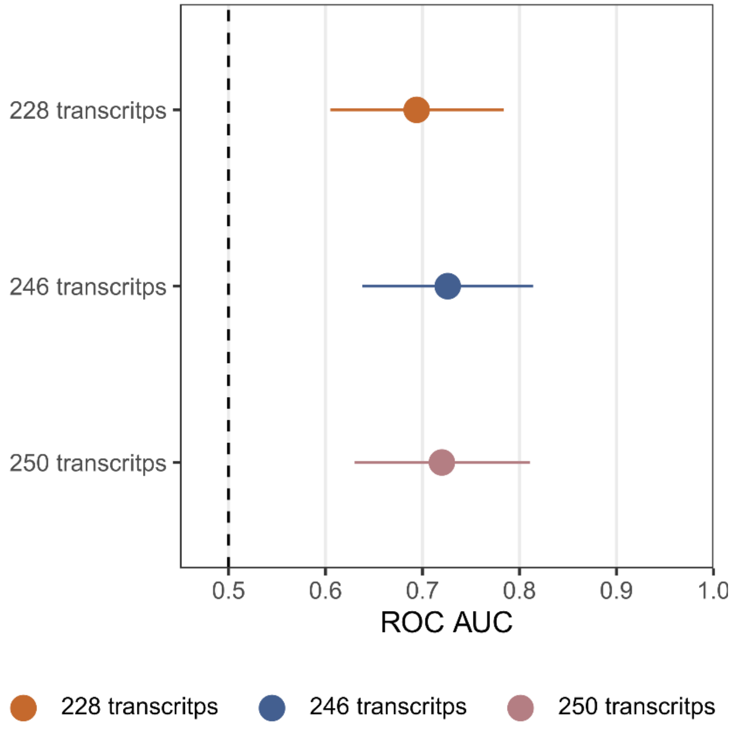

Supplement: Supplementary file 1 [file awag014_supplementary_data.zip › SupplementaryFigures.pdf]
