# Supplementary material for: Study of blood linear RNA nominates CD55 and DLD as early-stage biomarkers for Parkinson’s sisease
Source: Brain. Author manuscript; Available in PMC 2026 Sep 18. (PMC13584185; doi:10.1093/brain/awag014)

Study of Blood Linear RNA nominates *CD55* and *DLD* as early-stage biomarkers for Parkinson's Disease

Aleksandra Beric,[1,2†] Sarp Sahin,[1,2†] Santiago Sanchez,[1,2] Zining Yang,[1,2] Ravindra Kumar,[1,2] Isabel Alfradique-Dunham,[3] Jessie Sanford,[1,2] Daniel Western,[1,2] Bridget Phillips,[1,2] John P. Budde,[1,2] Richard J. Perrin,[4] Paul T. Kotzbauer,[3] Joel S. Perlmutter,[3,5,6,7,8] Scott A. Norris,[3,5] Carlos Cruchaga,[1,2,3,9,10,11] Laura Ibanez,[1,2,3,9]

[†]These authors contributed equally to this work.

1- Department of Psychiatry, Washington University in Saint Louis School of Medicine, St. Louis, MO 63110
2- NeuroGenomics and Informatics Center, Washington University in Saint Louis School of Medicine, St. Louis, MO 63110
3- Department of Neurology, Washington University in Saint Louis School of Medicine, St. Louis, MO 63110
4- Department of Pathology & Immunology, Washington University in Saint Louis School of Medicine, St. Louis, MO 63110
5- Department of Radiology, Washington University in Saint Louis School of Medicine, St. Louis, MO 63130
6- Department of Neuroscience, Washington University in Saint Louis School of Medicine, St. Louis, MO 63110
7- Program of Physical Therapy, Washington University in Saint Louis School of Medicine, St. Louis, MO 63108
8- Program of Occupational Therapy, Washington University in Saint Louis School of Medicine, St. Louis, MO 63108
9- Hope Center for Neurological Disorders, Washington University in Saint Louis School of Medicine, St. Louis, MO 63110
10- The Charles F. and Joanne Knight Alzheimer Disease Research Center, Washington University in Saint Louis, St. Louis, MO 63108

11- Department of Genetics, Washington University in Saint Louis School of Medicine, St. Louis, MO 63110

Correspondence to: Laura Ibanez, PhD

Washington University in Saint Louis School of Medicine

4444 Forest Park Ave

Campus Box 8134

Saint Louis, MO 63110

ibanezl@wustl.edu

Running title: **Novel Parkinson's Disease biomarkers**

Keywords: Parkinson's disease; blood transcriptomics; CD55; DLD; biomarkers

Supplementary Figure 1. Differential expression analyses. Volcano plots showing results of differential expression analyses in A. PDBP (maroon), B. PPMI (dark blue), C. BioFIND (light yellow) and 4. WashU-MDC (dark rosy-brown)

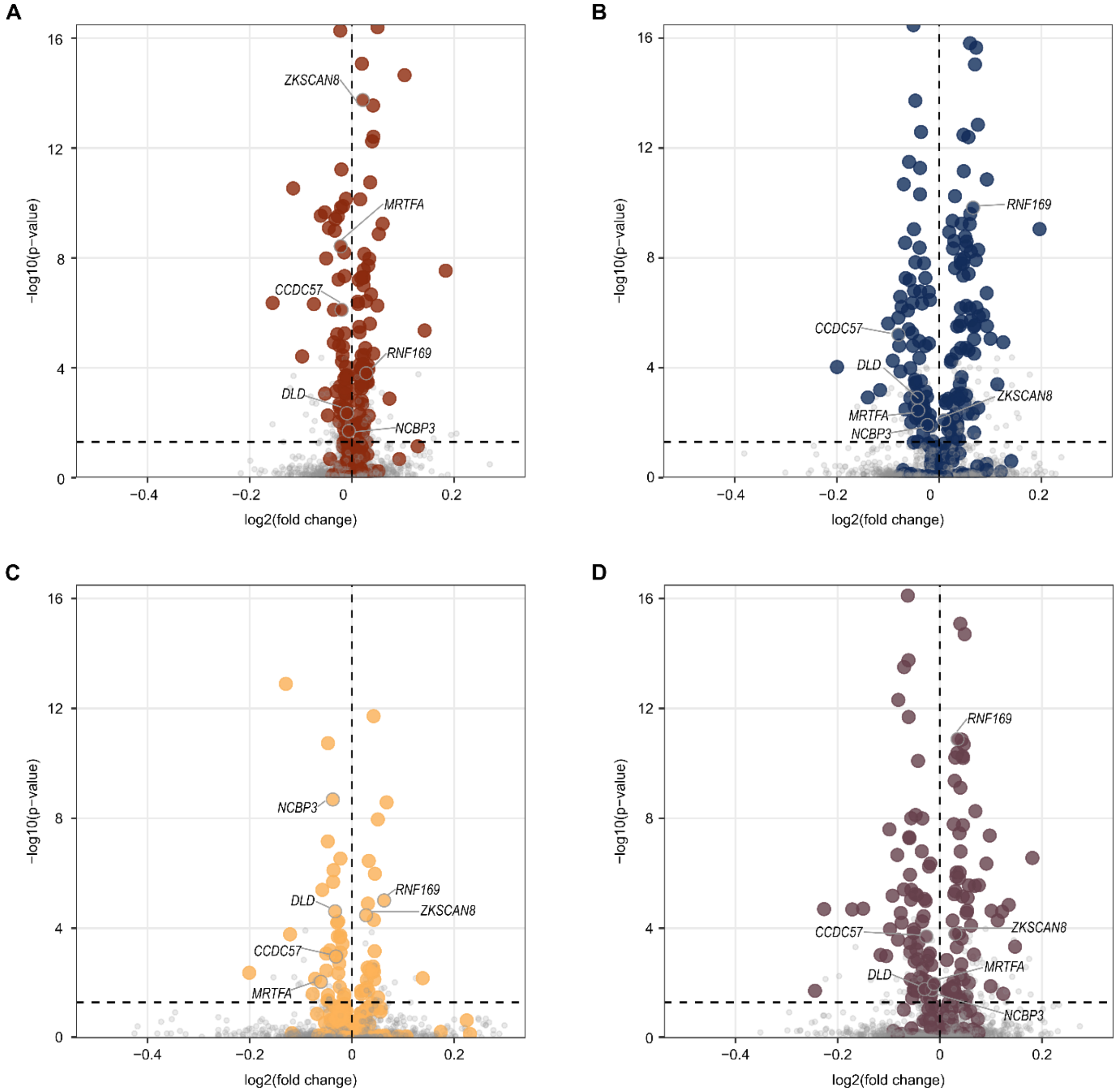

Supplementary Figure 2. Correlation.

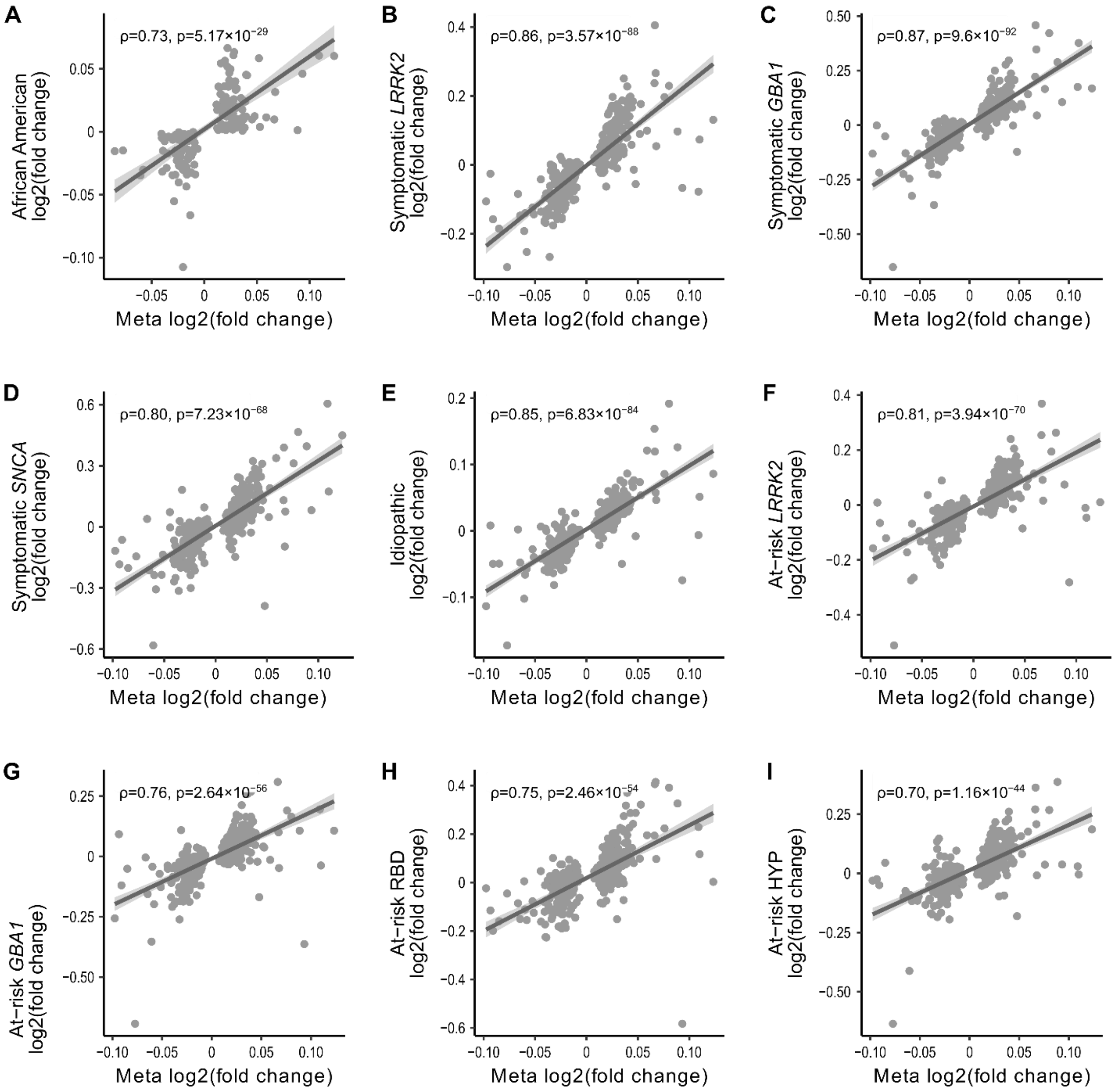

Supplementary Figure 3. Local Manhattan plots.

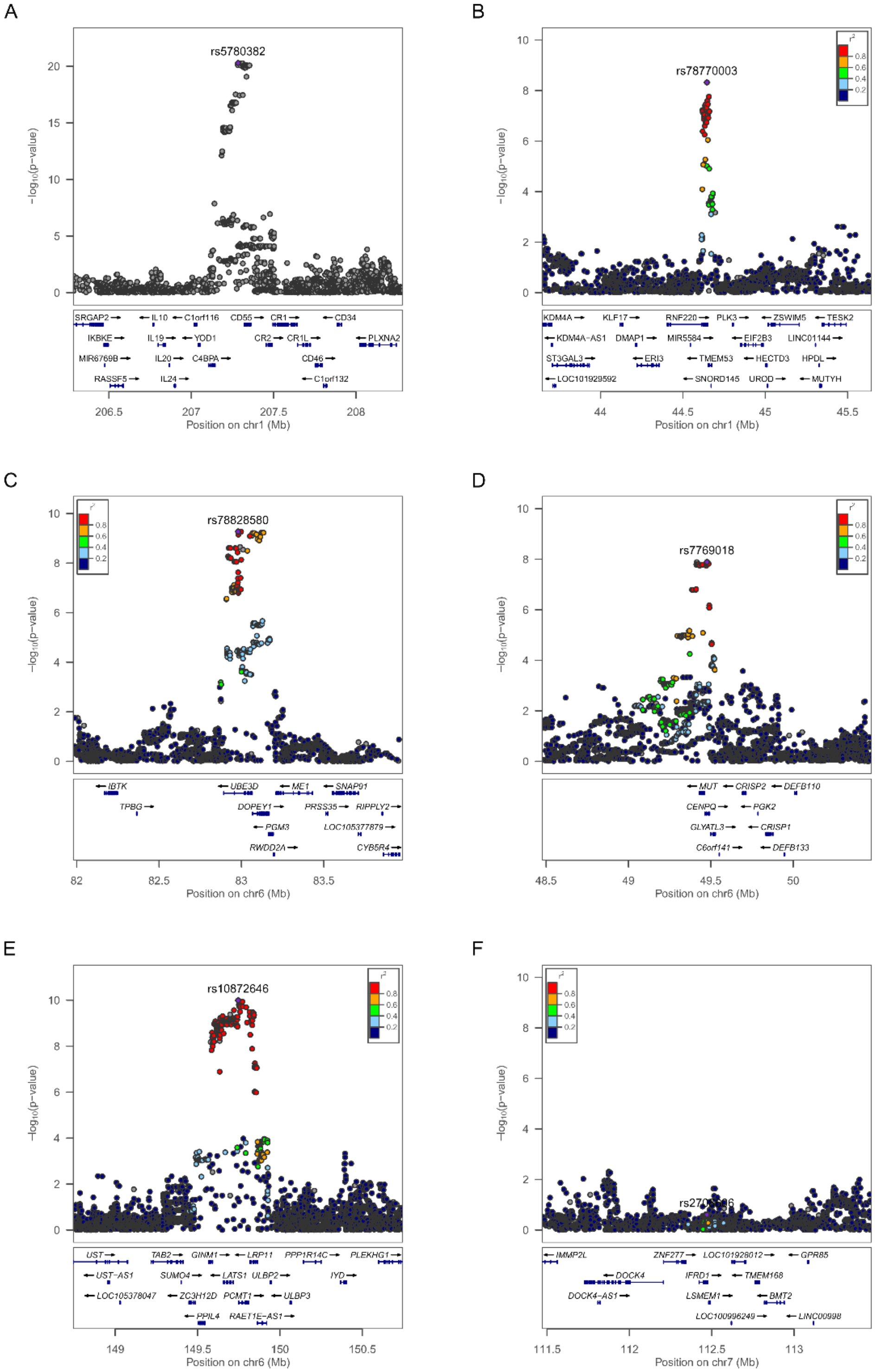

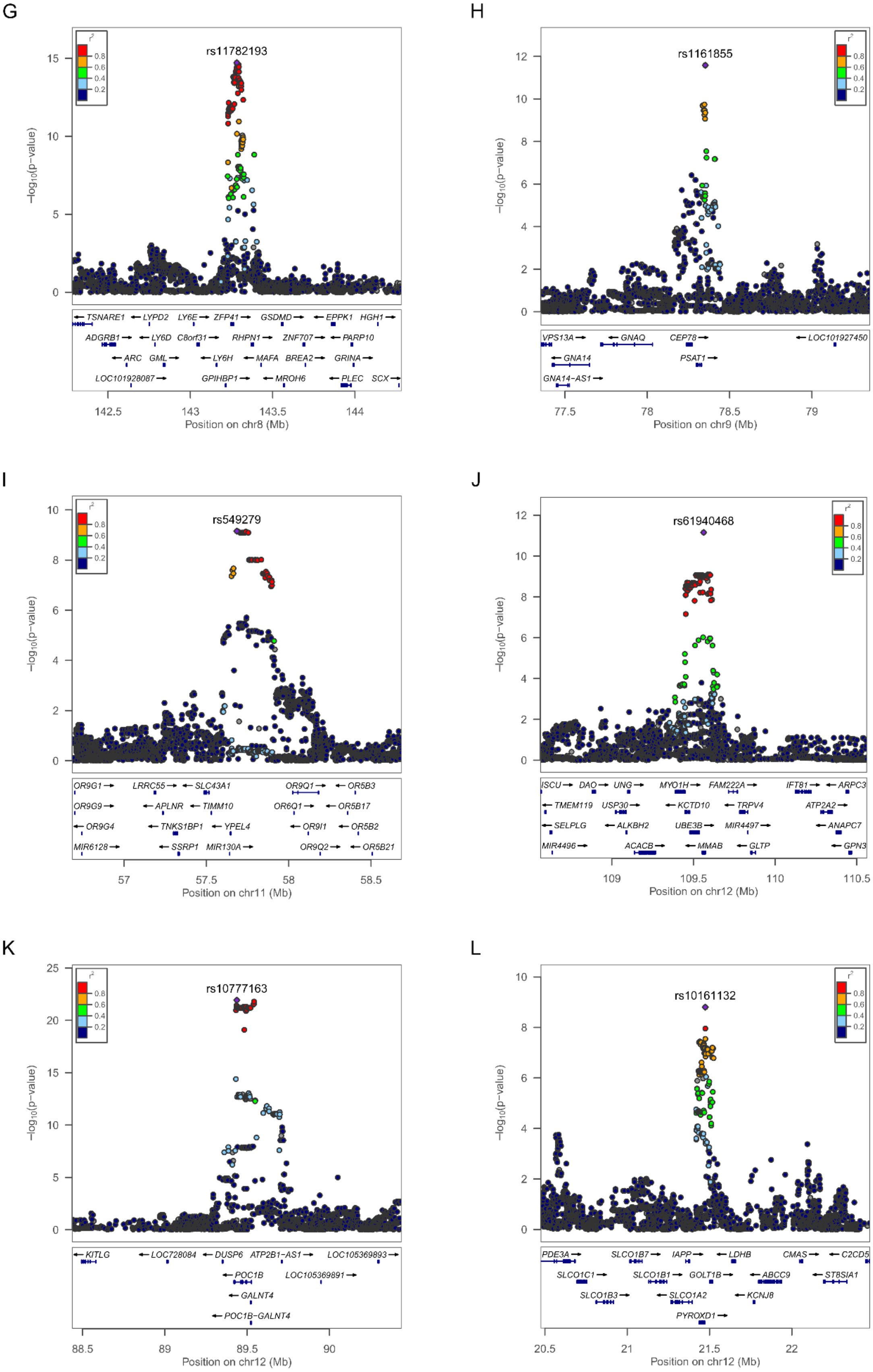
G
rs11782193
−log10(p−value)
Position on chr8 (Mb)
TSNARE1 LYPD2 LY6E ZFP41 GSDMD EPPK1 HGH1
ADGRB1 LY6D C8orf31 RHPN1 ZNF707 PARP10
ARC GML LY6H MAFA BREA2 GRINA
LOC101928087 GPIHBP1 MROH6 PLEC SCX
H
rs1161855
−log10(p−value)
Position on chr9 (Mb)
VPS13A GNAQ CEP78 LOC101927450
GNA14 PSAT1
GNA14−AS1
I
rs549279
−log10(p−value)
Position on chr11 (Mb)
OR9G1 LRRC55 SLC43A1 OR9Q1 OR5B3
OR9G9 APLNR TIMM10 OR6Q1 OR5B17
OR9G4 TNKS1BP1 YPEL4 OR9I1 OR5B2
MIR6128 SSRP1 MIR130A OR9Q2 OR5B21
J
rs61940468
−log10(p−value)
Position on chr12 (Mb)
ISCU DAO UNG MYO1H FAM222A IFT81 ARPC3
TMEM119 USP30 KCTD10 TRPV4 ATP2A2
SELPLG ALKBH2 UBE3B MIR4497 ANAPC7
MIR4496 ACACB MMAB GLTP GPN3
K
rs10777163
−log10(p−value)
Position on chr12 (Mb)
KITLG LOC728084 DUSP6 ATP2B1−AS1 LOC105369893
POC1B LOC105369891
GALNT4
POC1B−GALNT4
L
rs10161132
−log10(p−value)
Position on chr12 (Mb)
PDE3A SLCO1B7 IAPP LDHB CMAS C2CD5
SLCO1C1 SLCO1B1 GOLT1B ABCC9 ST8SIA1
SLCO1B3 SLCO1A2 KCNJ8
PYROXD1

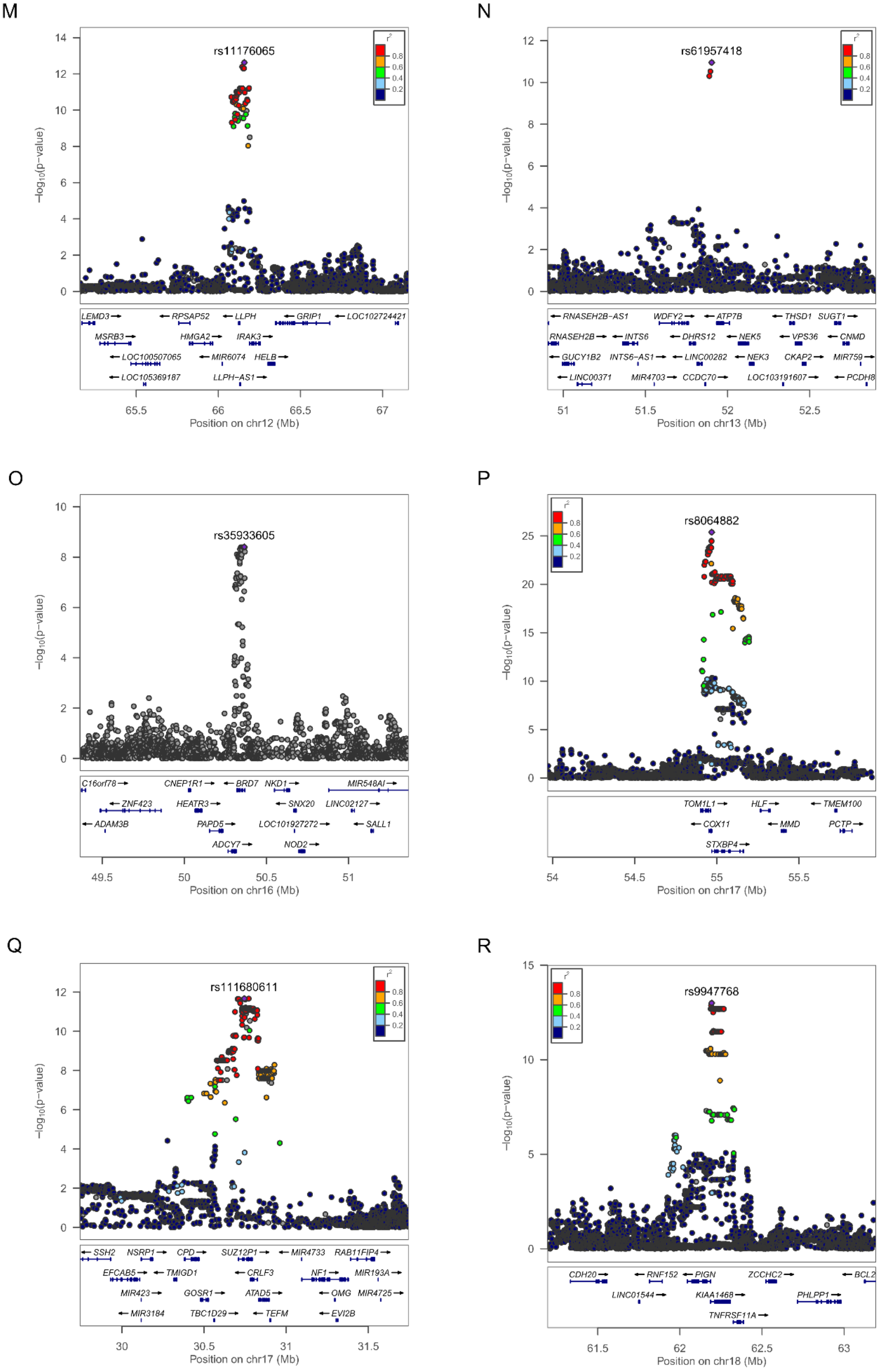
M
rs11176065
−log10(p−value)
Position on chr12 (Mb)
N
rs61957418
−log10(p−value)
Position on chr13 (Mb)
O
rs35933605
−log10(p−value)
Position on chr16 (Mb)
P
rs8064882
−log10(p−value)
Position on chr17 (Mb)
Q
rs111680611
−log10(p−value)
Position on chr17 (Mb)
R
rs9947768
−log10(p−value)
Position on chr18 (Mb)

Supplementary Figure 4. Feature Selection. Model performance in the training population for increasing KLD cutoff values. The Y-axis represents the AUC of each model, and the X-axis represents the KLD value used as threshold, with models selected for further analyses highlighted in orange.

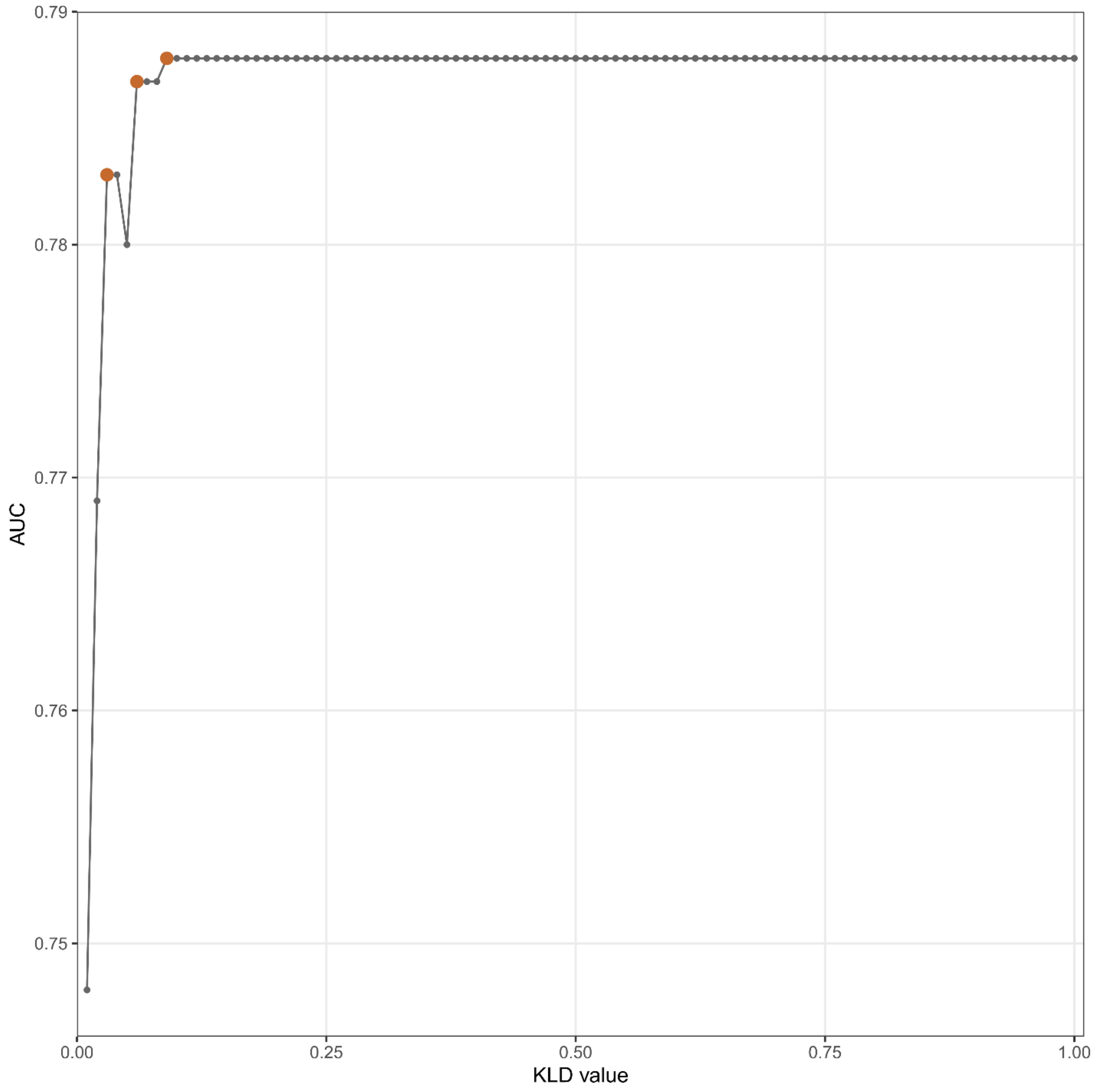

Supplement: Supplementary file 1 [file NIHMS2207765-supplement-supplemental_data.pdf]
